# Trunk spines of trees: a physical defence against bark removal and climbing by mammals?

**DOI:** 10.1093/aob/mcac025

**Published:** 2022-02-24

**Authors:** Théodore Lefebvre, Tristan Charles-Dominique, Kyle W Tomlinson

**Affiliations:** 1 Center for Integrative Conservation, Xishuangbanna Tropical Botanical Garden, Chinese Academy of Sciences, Menglun, Yunnan, China; 2 University of Chinese Academy of Sciences, Beijing, China; 3 Institute of Ecology and Environmental Sciences, CNRS UMR 7618, Sorbonne University, Paris, France; 4 Center of Conservation Biology, Core Botanical Gardens, Chinese Academy of Sciences, Menglun, Yunnan, China

**Keywords:** bark feeder, debarking, defence, function, mammal, morphological syndrome, trunk spine

## Abstract

**Background and Aims:**

The defensive role of spines has previously been related to leaves, young shoots and reproductive organs. However, some woody species harbour spines on their trunks where none of those organs are present. Several explanations are plausible: they could be (1) climbing aids, (2) remnants from defence of leaves or reproductive organs during an earlier development phase, or (3) an as-yet undescribed defence. Here we investigate whether they could play a role against either bark feeding or preventing climbing animals accessing food resources in the tree canopy.

**Methods:**

We described 31 woody species with spines on their trunk, growing in a botanical garden, to test whether morphological strategies could be identified and suggest what could be their most likely function. As testing their function is difficult experimentally for large pools of species, we performed virtual experiments to evaluate the potential roles of trunk spines against bark removal and climbing animals of different sizes. We then compared for each species and their confamilial non-spiny species the nutritional profiles of leaf, bark and reproductive organs to test whether trunk spines were associated with a nutritious organ (more likely targeted by herbivores).

**Key Results:**

We identified four morphological syndromes of trunk spines. Two corresponded to already known functions (anchorage for lianas and crown defence against large ground mammals), and two strategies are newly described trait syndromes with traits suggesting a defence against bark feeding and climbing mammals. By simulation, we show how each strategy could translate into defence against debarking and prevent herbivores from climbing.

**Conclusions:**

We identified trunk spine strategies and the criteria to classify them, their most likely function and the likely feeding mode and size of animal against which different trunk spine strategies may be effective. We discuss further perspectives for testing their function and their ecological significance.

## Introduction

**Fig. 1.**
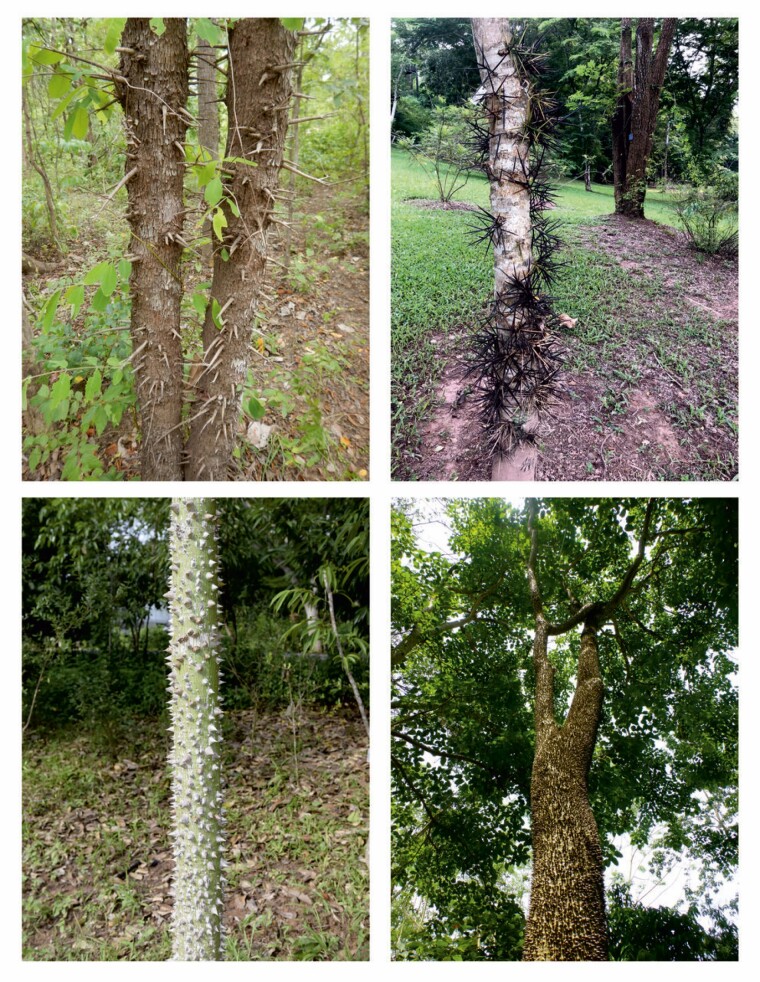
Pictures of spiny trunk species illustrating the two spiny syndromes Thorny trunk (top) and prickly trunk (bottom) related to new functions of spinescence. *Cratoxylum cochinchinense* (top left), *Gleditsia microphylla* (top right), *Ceiba speciosa* (bottom left) and *Hura crepitans* (bottom right).

Spines on plants are widely distributed across plant families (Charles-Dominique *et al.*, 2016) and have evolved from several plant organs (Bell and Bryan, 2008; Armani *et al.*, 2020). There is strong evidence to suggest that spines have evolved predominantly as defences against mammalian herbivores (Cooper and Owen-Smith, 1986; Charles-Dominique *et al.*, 2016). Spines are also used as anchors for lianas (Putz, 1990; Grubb, 1992; Isnard and Rowe, 2008) and have been proposed to play a role against water losses in cacti (Nobel, 2002) or slowing down caterpillars when climbing on stems (Yamazaki *et al.*, 2014; Kariyat *et al.*, 2017). The different syndromes of spiny plants have barely been explored and we do not yet have a complete synthesis of their function based on their morphological attributes. According to the class of animal that is targeted, the plants may display very contrasted traits as the defence, according to the organs being targeted by mammals (Gowda, 1996), according to plant ontogeny (Armani *et al.*, 2019), or depending on the maximal height that animals can reach (Burns, 2014). Most research to date has evaluated how spines defend against ground-dwelling mammals (Belovsky *et al.*, 1991; Gomez and Zamora, 2002; Milewski and Madden, 2006) while only a few studies have tested how spines could affect climbing mammals (Cooper and Ginnett, 1998; Liu *et al.*, 2020) or invertebrate herbivores (Yamazaki *et al.*, 2014; Kariyat *et al.*, 2017). In most of these studies, spinescence is either shown or assumed to defend soft edible parts such as leaves or reproductive organs (Grubb, 1992; Ronel and Lev-Yadun, 2012; Xu *et al.*, 2020). Interestingly, some tree species sometimes recruit spines on their trunk in positions where no soft organs are present (Fig. 1). Janzen and Martin (1982), observing these spines in South America, suggested that these might be anachronistic defences against the extinct megafauna, including giant sloths, and might additionally prevent rodents from climbing, but their hypothesis has remained untested.

Comparing the vertical distribution of spines on species from different regions, Burns (2016) found that spines are expressed within the plant body at heights that match the browse range of the dominant ground herbivores. However, in trees with trunk spines, some species produce spines only at the base of the plant (up to 3 m) while others have spines covering the trunk up to 20 m (Fig. 1), suggesting that trunk spines might be protecting trunks against terrestrial mammals in some contexts and against climbing animals in other contexts.

Spiny plants that defend their foliage have been shown to have highly nutritious foliage (Rafferty *et al.*, 2005; Tomlinson *et al.*, 2016). By analogy, we could expect tree species defending their trunk from debarking to have a nutritious bark, as a non-nutritious organ would be less likely to be targeted by herbivores. Bark removal by mammals is commonly observed across all continents and for a large range of clades, including Primates (Di Bitetti, 2019), Rodentia (Gill, 1992*b*; Baxter and Hansson, 2001), Artiodactyla (Klich, 2015; Nicodemo and Porfírio-da-Silva, 2019), Lagomorpha (Gill, 1992*b*; Baxter and Hansson, 2001), Hyracoidea (Barry and Shoshani, 2000), Diprotodontia (Stephens *et al.*, 2006), Perissodactyla (Steinheim *et al.*, 2005) and Proboscidea (Joshi and Singh, 2008; Ihwagi *et al.*, 2009). We know that debarking can play a very important ecological role for both mammals and tree populations (Yeaton, 1988; Yokoyama *et al.*, 2001; Ihwagi *et al.*, 2009). On the animal side, only a few animals are known specialist bark feeders, such as porcupines that can use bark as their main food source (Akram *et al.*, 2017). However, for non-specialists, debarking can play a critical role in their survival as bark provides food in the non-growing season (winter at high latitudes and dry seasons in tropical and subtropical latitudes) (Verheyden *et al.*, 2006; Di Bitetti, 2019; Nicodemo and Porfírio-da-Silva, 2019) when other food sources are scarce. For example, bark can constitute >10 % of the diet of *Cervus elaphus* in Europe (Verheyden *et al.*, 2006). On the plant side, the removal of a ring of bark around the trunk can kill large trees and cascade into tree community changes (Noel, 1970; Ihwagi *et al.*, 2009; Vanak *et al.*, 2012). Debarking is affected by several plant traits, including bark thickness and toughness and branches that act as obstacles for debarking (Gill, 1992*b*; Kuiters *et al.*, 2006; Vospernik, 2006; O’Connor *et al.*, 2007); however, the role of spines as defences against debarking has not been evaluated.

In this paper, we investigate possible functions of spines that occur on the trunks of woody species. We hypothesized that spines present on the trunk could be involved in four separate functions: defending the trunk bark against ground-dwelling mammals (h1); defending canopy resources (leaves, bark or reproductive organs) against climbing mammals (h2) (Janzen and Martin, 1982); defending leaves and/or reproductive organs against ground-dwelling mammals in early developmental stages within their reach (h3); and used by lianas as tools for anchoring the plant to a support (h4). We expected: (1) plants defending their trunk against debarking by ground-dwelling mammals (h1) to grow spines at the base of trunk and to maintain their spines over time to maintain the defence on a growing trunk; (2) plants defending their canopy against climbing mammals (h2) to develop their spines in the canopy and on main branches above the heights that ground-dwelling mammals can reach, and to develop high densities of spines that could slow down a climbing animal; (3) plants defending the organs in their crowns against ground-dwelling mammals (h3) to produce their spines near and simultaneously to those organs, with no need to maintain these defences once the protected organs have been pruned, and only within the reach of ground-dwelling mammals; and (4) lianas that use their spines as an anchorage tool (h4) to have curved spines produced on young non-self-supporting trunks, along the entire length of the trunk, with no additional new spines after the stem elongation phase is complete.

We analysed the traits of 31 tropical woody species with spines on their trunk, using the living collections of Xishuangbanna Tropical Botanical Garden (XTBG), situated in Yunnan Province, China. First, we analysed whether species with trunk spines have common morphological syndromes that could inform about their likely function (timing, location, association with organ, maintenance and densities). Second, we mapped spine placement on the trunks and used computer simulation to evaluate whether the identified syndromes differed in their potential to defend against either debarking or climbing. Finally, we compared the nutritiousness (nitrogen concentration, total phenols and inner bark thickness) of leaf and bark, and the attractiveness of flowers and fruits from 31 spiny species and 25 non-spiny species, to test whether spiny trunk species were defending more nutritious organs than non-spiny species, and whether there were differences in nutritiousness among the identified syndromes.

## MATERIALS AND METHODS

### Species selection

We described 31 species of woody species with spines on their trunk (from 25 genera and 16 families; Supplementary Data Table S1) in the Xishuangbanna Tropical Botanical Garden, an institute of the Chinese Academy of Sciences (XTBG-CAS), located in Menglun, Yunnan, China (21°55′38″N, 101°15′6″E). These species originated from different tropical areas around the world (POWO, 2019) and were described on mature individuals. When several individuals were available in the garden, we described the individual with the densest spine cover on the assumption that they could relate better to spine expression in an environment where these species are exposed to herbivores, since allocation to spines increases under herbivore pressure (Milewski *et al*., 1991). These observations were complemented by descriptions of two species, *Cratoxylum cochinchinense* and *Olax scandens*, from individuals in Chatthin Wildlife Sanctuary, in Myanmar (23°36′N, 95°32′E). We did not consider non-woody plants that possess spines on their stems, such as Arecaceae and Cactaceae. We then compared the nutritiousness of organs of the 31 spiny species with their 25 non-spiny confamilial species present in the garden (Supplementary Data Table S1).

### Morphological analysis

**Fig. 2.**
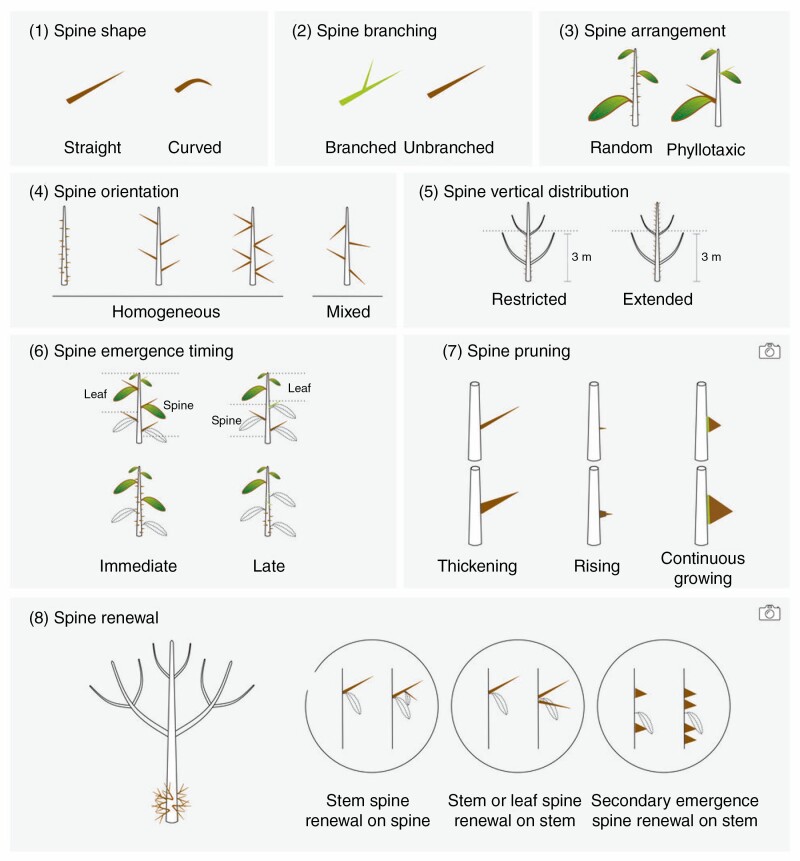
Illustration of the new variables and related traits of trunk spinescence.

The morphology of each spiny species was described according to 12 morphological variables. These variables were selected to discriminate the four functions that were hypothesized for explaining the presence of spines on the trunk of trees. They describe the temporal and spatial distribution of spines and their individual effect over the four possible functions (Fig. 2, Table 1; Supplementary Data Fig. S2). Spine types and anatomical origin of spines (Bell and Bryan, 2008) were recorded but used only for interpretation and not in analysis, to avoid confounding functional attributes with their anatomical origin.

**Table 1.** List of variables and related assumptions of trait significance used in the morphological analysis of spinescence

No.	Trait	Trait states	Description	Assumption	Citation
1	Main stem habit	Self-supporting/non-self-supporting	Lianas and semi-self-supporting plants have, at least in part of their development, main stems that are not self-supporting.	Only non-fully self-supporting plants require anchorage tools.	Rowe and Speck, 2005
2	Spine density	Density (number cm²)	Density of spiny appendages on the trunk at breast height.	The density of spines on old trunks is expected to be low if spines defend an organ in the crown or serve as an anchorage tool.	
3	Spine length	Length (cm)	Length of the spine.	Shorter spines could prevent the animal from inserting paws and teeth; long spines could create an obstacle to body progression and head.	
4	Spine shape	Curved/straight/straightened	Spines can be curved, fully straight, or curved in a primary developmental phase and then straighten (this variable is called straightened) and straightened due to cambial growth. Straightened spines are curved when first they harden off but continue to grow over years and become straight as they get older.	Curved spines are hypothesized to be more efficient for anchoring the plant to a support.	Isnard and Silk, 2009
5	Spine branching	Branched/unbranched	Some thorns can branch immediately into lateral spiny branches.	Branched spines are expected to extend the radius of impact of individual spines.	New variable
6	Spine orientation	Homogeneous/mixed	Spines can be developed in various directions, allowing spines to cross each other, or following a specific organization, such as being perpendicular to the trunk.	Mixed spines can create a more complex defence matrix than non-mixed spines.	New variable
7	Spine arrangement	Phyllotaxic/random	Spines are phyllotaxic when expressed only at the node location and considered random when also expressed on the internodal stem segments.	Randomly distributed spines can offer a more homogeneous covering of the trunk surface, while phyllotaxic spines have location constrained by nodes.	New variable
8	Spine emergence timing	Immediate/late	Spines are immediate when they are associated in time and space with leaf presence and late if they develop after the leaves have been pruned.	Spines are expected to be associated spatially and temporally with leaves when protecting them against mammals.	New variable
9	Spine pruning	Maintained/pruned	Spines can be maintained for an extended period on the trunk by continuous growth of the structure (cork spine), by thickening, or by rising.	Maintained spines are expected to be associated with a defensive function associated with the trunks.	New variable
10	Spine renewal	Renewal/no renewal	Spines continue to be recruited at internodes or nodes on the stem after the associated leaves have dropped off (renewal). Spines are not recruited on stem after nodal leaves are pruned.	Spine renewal is expected to make it possible to keep spine density high enough on old stems to protect the trunk or to protect the canopy against climbing mammals.	New variable
11	Spine vertical distribution	Extended/restricted	Vertical distribution of spines is considered extended when spinescence is expressed on the trunk above 3 m height and restricted otherwise.	Most ground mammals cannot reach over 3 m height. Spine distribution then informs about whether spines are specialized in defending against ground mammals.	Burns, 2014
12	Spine type	Thorn/leaf/stipule/prickle/cork	Thorns are modified branches, leaf spines are modified petioles, stipular spines are modified stipules, prickles and cork are spines emerging on the internodal stem segment from epidermic and phellogenic layer, respectively.	Used only for interpretation.	

Continuous variables were log-transformed. We then identified morphological syndromes using Ward’s clustering (Murtagh and Legendre, 2014) on morphological variables transformed to vary from 0 to 1 to remove their dimensionality and weight them equally. The contributions of each variable to the clustering were estimated with the correlation ratio method (Kleyer *et al.*, 2012). Analyses were performed in R version 4.0.4 (R Core Team, 2021) using the vegan (Oksanen *et al.*, 2020) and ade4 (Dray and Dufour, 2007) packages.

### Evaluation of spine performance by simulation

We used simulations to evaluate the potential of trunk spines to restrict access to bark by bark-feeding mammals and impede movement of climbing mammals (Supplementary Data Fig. S2). We first mapped the spine distribution at the base of one trunk per species where the spine density was maximal, using 30-cm-long strings pinned vertically on the trunk every 2 cm around the stem. These strings separated strips of 2 cm width and 30 cm length in which the position and length of each spine were recorded. Spine coordinates were digitized using WebPlotDigitizer (v. 3.9) (Rohatgi, 2015) on pictures of all strips. The coordinates obtained were then collated to produce a 2D map of the trunk spine distribution for each species, which we used in simulations to test their impedance on debarking and climbing animals. We coded our simulation so that the left and right sides of the maps were merged to create a tor, as maps are a plane projection of a cylinder surface, but climbing and debarking herbivores have access to the whole cylinder.

We first simulated whether spines could prevent debarking mammals from accessing the trunk surface with their muzzles. We used a range of mouth sizes simulated by a square with a side ranging from 1 to 10 cm (with 1-cm increments). For each location on the stem, we then evaluated whether the herbivores could insert their mouth without encountering a spine. We considered that any spine in or within a 1-cm range from the area corresponding to the bite size could prevent an animal from inserting its mouth. Two additional effects are specific to plants with smaller diameters. First, a spine located perpendicular to the mouth insertion on the trunk might not affect the animal even if located in close proximity to the debarked area. Second, the animals are feeding on the surface of the stem (not cutting the wood inside the cylinder), so the curvature of the stem imposes a constraint on the insertion of teeth in bark. The moving window corresponding to the bite was therefore set not to exceed one-fourth of the stem circumference. After evaluating all positions that can be accessed by debarking animals across mouth sizes, we exported as output values the total proportion of bark that can be removed and the risk of ring debarking (calculated as the proportion of the stem that can be debarked continuously around the stem) for all mouth sizes for each plant species. These two measures were then compared across spiny species to analyse the potential of each spiny species group to impede debarking and ring-barking on their trunk.

Second, we evaluated whether spines could slow mammal climbing by affecting their capacity to insert their paws between spines on the trunk. We used a range of paw sizes simulated by a square with a side ranging from 1 to 10 cm (with 1-cm increments). All spines that were mapped were associated with a time penalty when the mammal path crossed a spine (penalty = 10). This value was chosen arbitrarily as no published data could be used to parameterize these effects. We then simulated an animal climbing that would choose the best available path on the trunk after an initial evaluation (this corresponded to a trained animal that had already experienced walking along the trunk). We did so by generating paths of increasing complexity: a straight line requires less time to cross than a more complex trajectory in the absence of spines. The first paths generated were straight lines. The time required to cross these paths was calculated as a function of distance and penalty due to encountered spines. The time required to cross the best path for the straight lines (less time) was retained and compared with a new set of paths in which the animal was allowed to change trajectory once. If a faster path was found within this new set (despite the longer path), a set of paths with two changes of trajectory was simulated and the procedure was repeated, and so on until further complexification of the animal path did not improve its speed in crossing the trunk segment. For every level of complexity of the path, we simulated 100 000 paths.

### Nutritional analysis of plant edible parts

The nutritional profiles of leaves and inner bark were estimated using the concentration of nitrogen (milligrams per gram) (Pérez-Harguindeguy *et al.*, 2013), the level of total phenols (Singleton and Rossi, 1965) and by measuring the inner bark thickness (millimetres). The two first variables describe the nutritional quality of bark and the third describes the quantity that is available.

Samples were collected in May 2017 (beginning of the wet season) and additionally in March 2019 for the inner bark (end of the dry season). We did not have permission to collect bark samples directly from the main trunk (destructive sampling), so sampled only secondary trunks with equivalent physiological properties (see reiteration in Barthélémy and Caraglio, 2007). The outer bark layer was not removed from the inner bark layer as it was always <1 mm in all sampled species. We sampled leaves (including petioles) on long horizontal branches at the periphery of the crown. Replications were done on one to six individuals according to availability in the garden (Supplementary Data Table S2). Several species could not be sampled for this analysis either because we were unable to access the leaves or because they had no leaves at the time of sampling: *Olax scandens*, *Zanthoxylum myriacanthum*, *Paliurus ramosissimus* and *Flacourtia rukam*. All samples were dried, ground and analysed (Singleton and Rossi, 1965; Pérez-Harguindeguy *et al.*, 2013) at the Public Technology Service Centre (Central Laboratory) of Xishuangbanna Tropical Botanical Garden.

The attractiveness of fruits and flowers was assessed for each species based on their morphological structure. The fruit attractiveness was classified as ‘high’ for species with fleshy, smelly and colourful fruits, ‘medium’ for species without fleshy fruits but with large structurally unprotected seeds, such as Fabaceae pods, and ‘low’ for species without fleshy fruits and with small seeds or anemochorous seeds. Flowers were classified according to the diameter of the corolla as small (≤2 cm) or large (>2 cm).

### Statistical analysis

We used Bayesian mixed effect models to compare the defensive performance of each syndrome as derived from the simulation experiments, and to compare their nutritional profiles. The statistical analyses were conducted with the brms package (Bürkner, 2017) in R version 4.0.5 (R Core Team, 2021). The simulation experiments were analysed assuming binomial errors for the bark removal trials (bounded between 0 and 100) and assuming Poisson errors for the climbing trials (bounded below at 0). Overdispersion was accounted for by including an observation level random effect (Harrison, 2014, 2015). The nutritional analyses were performed assuming log-normal errors for nitrogen, total phenol and inner bark thickness. The nutritional models included as additional random effects the sampling date (month) for bark analyses and the phylogenetic relatedness among species.

All model parameters were set with the default weakly informative priors (Gelman *et al.*, 2008) and were run using four chains of 35 000 iterations with a warm-up of 10 000 iterations, thinned every 10 steps, giving a total of 10 000 post-warm-up samples. Best models were selected using the approximate leave-one-out information criterion (LOOIC) on nested models (Vehtari *et al.*, 2017). The goodness of fit, *R*², was determined with a Bayesian method provided in brms, based on Gelman *et al.* (2019). Finally, a *post hoc* test was performed with the general non-linear hypothesis method from brms that evaluated the credible interval, set at 0.05, and the evidence ratio, which corresponds to the Bayes factor in two-sided hypotheses (Bürkner, 2017).

Phylogenetic trees were extracted from the megaphylogeny of vascular plants GBOTB.extended using the V.PhyloMaker package (Jin and Qian, 2019). Species names were checked using the Taxostand package (Cayuela *et al.*, 2012). The ultrametric phylogenetic trees were standardized using the method of Grafen (1989) from the ape package (Paradis and Schliep, 2019) and transformed into a correlation matrix of species pairwise distances, which was used in the mixed model as a phylogenetic random effect.

## RESULTS

### Morphological analysis of spiny trunk species

**Fig. 3.**
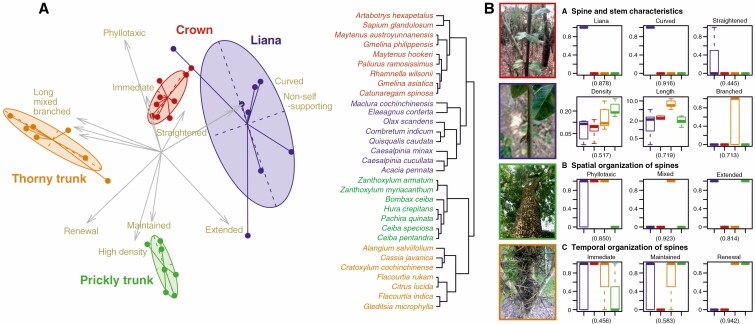
(A) Clustering of the 31 spiny species according to the 12 morphological variables (left). The clustering of species was done using Ward’s minimum variance clustering method and by keeping all eigenvectors. The representation is based on the two main components (PC1 = 29.97 % and PC2 = 23.25 %). (B) Pictures illustrating spinescence syndromes are from top to bottom: *Catunaregam spinosa*, Maclura cochinchinensis, *Hura crepitans* and *Gleditsia microphylla*. Contribution of each variable to the clustering and trait variation within syndromes determined with the correlation ratio method (right). The correlation ratio is indicated in parentheses under the variable graph. Qualitative variables indicate a presence (1) or an absence (0). The spine density and length are plotted with a log scale.

The clustering analysis of morphological traits found four main morphological syndromes (Fig. 3; Supplementary Data Tables S3–S6).

#### Prickly trunk syndrome (see Hura crepitans and *Ceiba speciosa*, Fig. 1).

This included species with a self-supporting trunk covered by cork-derived spines (suber spines) on the internodal section of the stem, and additionally, with prickles (epidermic spines) for *Zanthoxylum* species. Cork spines can be produced on old trunks (with spines emerging late in development). Prickle dimensions could be increased due to the addition of suber layers below its base (see rising mechanism; Fig. 2(7); Supplementary Data Fig. S1E, F). Cork spines are completely produced from suber, including their sharp tips (Fig. 2(7); Supplementary Data Fig. S1A, B). The spines were distributed homogeneously on the trunk, with the apices pointing perpendicularly to the trunk and were typically straight, unbranched, of medium size (mean = 2.2 cm) and with high densities on the trunk (mean = 2500 spines m²). For almost all species in the syndrome (except *Bombax ceiba*), spines were also established high on the trunk and main branches (above 3 m), but with a lower density over 2–3 m height (except for *Hura crepitans*).

#### Thorny trunk syndrome (see *Cratoxylum cochinchinense* and *Gleditsia microphylla*, Fig. 1).

This was characterized by species establishing long thorns (lateral branches modified as spines) produced together with leaves on a self-supporting trunk or, more rarely, after the leaves had senesced (e.g. *Cratoxylum cochinchinense* and *Cassia javanica*). In all species, recruitment of thorns continued after the primary axillary leaf had senesced. In many species, the spines were thickened over years via cambial growth (Fig. 2(7); Supplementary Data Fig. S1G, H). These spines derived from stems only developed at nodal positions, so their location was constrained by the plant phyllotaxy. Spines were straight, sometimes branched, long (mean = 8.9 cm), with medium to high density (mean = 1800 spines m²), and were not all perpendicular to the trunk as they can emerge laterally on previous thorns. In all species spines only occurred below 2–3 m height.

#### Crown syndrome.

This included species establishing thorns or stipular spines simultaneously with leaves, on young self-supporting axes. Most spines were pruned after a few years (compared with decades for the previous syndromes). The spines emerged at nodes (phyllotaxic), were straight and unbranched, were of medium size (mean = 2.7 cm) and were expressed at low to medium density (mean = 800 spines m²), perpendicularly to the trunk. Spines were not expressed in this syndrome above 2–3 m height. *Sapium glandulosum* differs from other members of the syndrome because of the timing of spine production, with thorns being made by ramifying short side branches after leaves are pruned.

#### Liana syndrome. 

This includes species with non-self-supporting stems with either immediate prickles or thorns, produced simultaneously with leaves, or late thorns or spines produced from secondary thickening of branch bases or leaf parts. Spines were established at all heights along the entire trunk. The spines were either phyllotaxic (thorns and leaf spines) or randomly distributed in internodal sections (prickles), and all species possessed curved spines (*Maclura cochinchinensis* and *Elaeagnus conferta* had both curved and straight spines), unbranched, of small to medium size (mean = 2 cm). Spines were displayed on the trunk at low to medium density (mean = 1300 spines/m²), and were perpendicular to the main stem. *Elaeagnus conferta* differs from other members of the syndrome because thorns are recruited on older trunks and developed only within a few metres from ground level.

### Defence performance simulations of spiny syndromes

#### Trunk spines as a defence against bark removal.

**Fig. 4.**
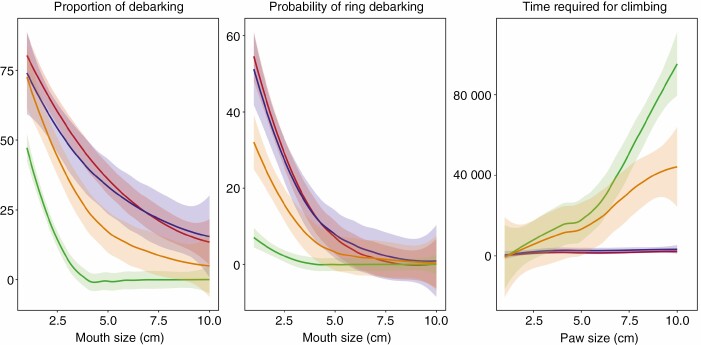
Simulated effect of spines on the capacity of mammals to debark and climb trees for each syndrome (see Supplementary Data Table S7 for raw values). The level of defence was expressed as the proportion of bark removed, the probability of ring debarking and the slowing down of mammals by preventing them from inserting their paws between spines. Computer simulations were performed on a range of mammals with a mouth or paw ranging from 1 cm × 1 cm to 10 cm × 10 cm. Posterior predictive parameters and multiple comparison parameters estimated from the Bayesian models are referenced in the Supplementary Data Tables S8 and S9.

In our simulations, access to bark is significantly lower in the prickly syndrome compared with other syndromes for all herbivore sizes (Fig. 4; Supplementary Data Tables S7–S9). The thorny syndrome defended better than the liana and crown syndromes against large herbivores. The risk of ring-barking was significantly reduced against small herbivores in the prickly and thorny syndromes compared with the liana and crown syndromes. All syndromes reduced the risk of ring-barking against large herbivores. The liana and crown syndromes did not differ significantly from each other in terms of available area for debarking and risk of ring-barking (Fig. 4; Supplementary Data Tables S7–S9).

#### Trunk spines as a defence against climbing herbivores. 

Our simulations suggest that the prickly syndrome had the most negative impact on climbing mammals, followed by the thorny syndrome, and both had a greater effect on large mammals compared with the liana and crown syndromes (Fig. 4; Supplementary Data Tables S7–S9). The liana and crown syndromes performed similarly (Fig. 4; Supplementary Data Tables S7–S9).

### Nutritiousness profiles of edible parts

**Fig. 5.**
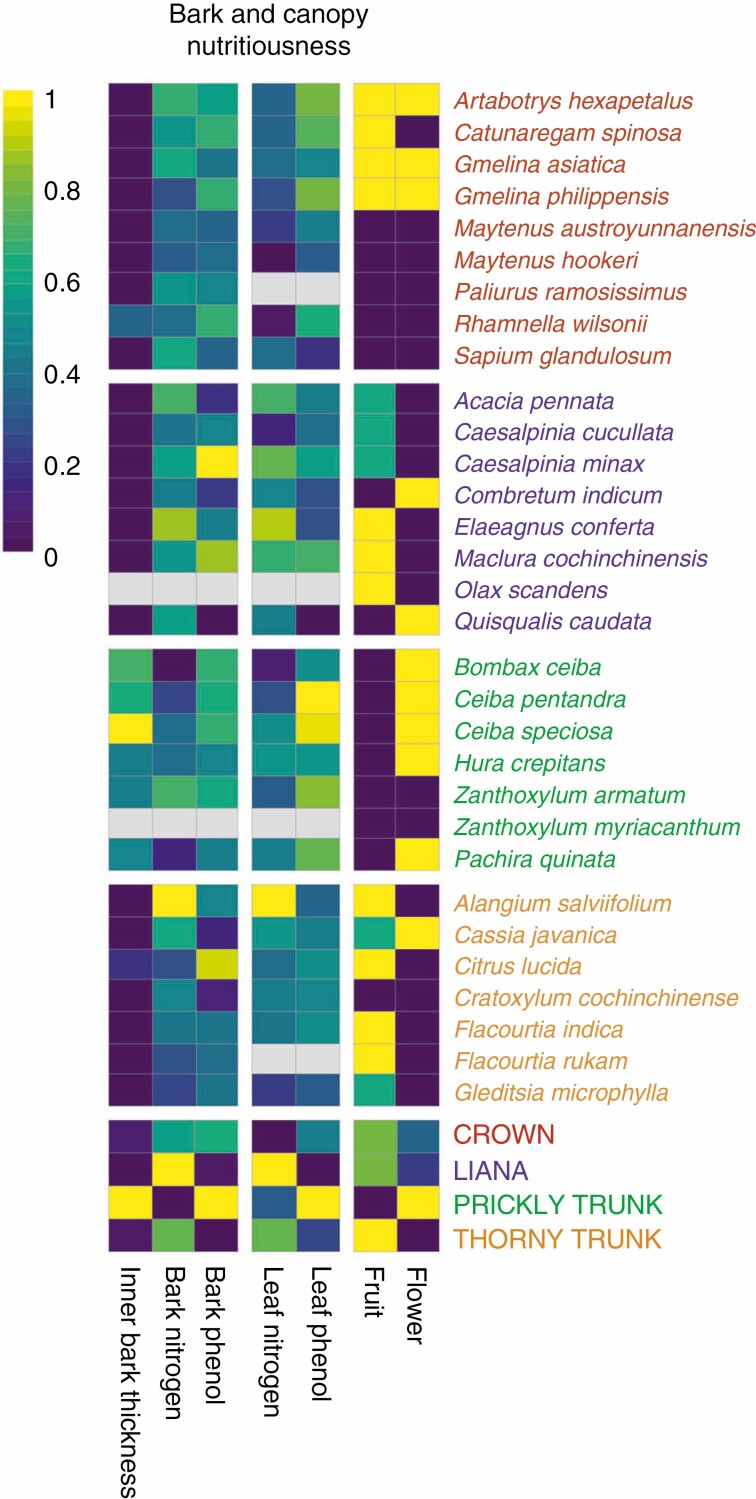
Nutritional profiles for the 31 spiny species and spinescence syndromes. High values in the gradient (yellow) indicate a more likely nutritious organ (e.g. yellow for leaf total phenols implies a low level of phenol). All continuous variables have been log-transformed and scaled from 0 to 1 to reveal their relative differences (see Supplementary Data Table S2 for raw values). Values for each species are based on the mean of the fitted values from the posterior predictions of models.

The leaves of species with prickly trunks had significantly lower concentrations of phenols compared with the thorny trunk and liana syndromes (Fig. 5; Supplementary Data Fig. S3, Supplementary Data Tables S2 and S10–S12). Bayes factors confirmed that prickly trunks had lower phenol concentration in their leaves than species from the crown syndrome and compared with their non-spiny confamilial species but did not differ significantly from those groups (Fig. 5; Supplementary Data Fig. S3, Supplementary Data Tables S2 and S10–S12). Results also indicate that spiny lianas had higher leaf nitrogen than non-spiny lianas and species from the crown syndrome (Fig. 5; Supplementary Data Fig. S3, Supplementary Data Tables S2 and S10–S12). Other syndromes were not significantly different from each other with respect to phenols and nitrogen content (Fig. 5; Supplementary Data Fig. S3; Supplementary Data Tables S2 and S10–S12). Bark content did not significantly differ between syndromes in terms of total phenols and nitrogen contents (Fig. 5; Supplementary Data Fig. S3, Supplementary Data Tables S2 and S10–S12). However, the prickly trunk syndrome had significantly more inner bark (at least 2 times thicker) than all other syndromes (Fig. 5; Supplementary Data Fig. S3, Supplementary Data Tables S2 and S10–S12). Finally, the species with prickly trunk syndrome had large attractive flowers (excepted *Zanthoxylum* sp.) and fruits with a low risk of predation and did not have adaptations to seed dissemination by non-flying mammals; the thorny syndrome had mostly small flowers and mammal-dispersed fruits (Fig. 5; Supplementary Data Table S13). The liana and crown syndromes had a mixture of fruit traits (Fig. 5; Supplementary Data Table S13).

## Discussion

We identified four morphological syndromes of woody plants with spines on their trunks (Fig. 3). Two of them had spines with a well-known function, either protecting leaves and reproductive organs from ground-dwelling mammals (referred to as the crown syndrome) (Grubb, 1992), or using curved spines as anchorage tools (referred to as the liana syndrome) (Isnard and Silk, 2009). In the crown syndrome, spines develop simultaneously and in proximity with leaves (Gowda, 1996), within the height range of ground-dwelling mammals (Burns, 2014), and they do not possess reinforcing mechanisms and disappear within a few years after leaves have senesced. In the liana syndrome, spines are curved (Isnard and Silk, 2009) and develop simultaneously and in proximity with leaves, and are produced on non-self-supporting stems, even over the height reachable by ground-dwelling mammals. In most species the spines are maintained over time, maintaining the anchorage function (Isnard and Silk, 2009), but also possibly contributing to plant defence. Results from the two other syndromes suggest that they have a function specifically associated with trunks of trees (Figs 1 and 3). These syndromes produce new spines on their trunks (referred to as ‘trunk spines’) after leaves have senesced, following the range of heights of ground-dwelling mammals in the thorny syndrome and sometimes until the tip of the trunk for the prickly syndrome (Figs 1 and 3). Simulations, in accordance with morphology, suggested that trunk spines of the thorny syndrome were likely defending against medium to large ground-dwelling bark-feeding animals, whereas the trunk spines of the prickly syndrome were likely defending against small to large ground-dwelling bark-feeding and climbing animals (Fig. 4). Thorny trunk species develop a medium density of long spines displayed in clusters on the trunk, at its base (2–3 m), while prickly trunk species typically develop a high density of medium conical cork spines (in the case of *Zanthoxylum* spp. prickles are also established) covering the whole trunk surface at the base of it, and sometimes up to the top of trees, whether at a similar density or lower density over the height reachable by ground mammals (excepted for *Bombax ceiba*, which had spines only on the first 2–3 m). Cork trunk spines are derived from the phellogen in all the species in our sample, similarly to a developmental pathway described in the genus *Rosa* (Schweingruber *et al.*, 2006). These are able to maintain a high density of spines on old trunks and branches despite stem secondary growth, whereas epidermic structures (prickles) cannot as they are only produced during primary growth. Both syndromes continue to recruit new spines on the trunk late in plant development long after leaves and fruits have senesced. Below, we discuss how the morphological syndromes, the organ nutritiousness and the simulation of effects could provide complementary information for identifying the function and the type of herbivore that is likely targeted by the defence.

### Using plant architecture to infer the possible function of spines

A large body of literature shows that herbivores with different feeding modes select for different plant defence strategies. While plants are structurally defended against insects with specific leaf ornaments (leaf cuticles, hairs, etc.; Kariyat *et al.*, 2018), their defence against large animals generally requires transformation of the whole plant body. For instance, plants growing in environments where large herbivorous birds are the dominant herbivores possess divaricate architecture with flexible stems that have strong tensile strength and are effective against the plucking feeding motion of birds (Bond *et al.*, 2004). Plants growing in open habitats such as savannahs and Mediterranean shrublands where mammalian herbivores are present possess sharp spines and cagey structures that limit the ability of mammals to cut off edible parts (Milewski *et al.*, 1991; Charles-Dominique *et al.*, 2017). The vertical height to which spines are expressed in many woody species matches the reach of native terrestrial herbivores (Burns, 2014), and it is apparent that spines are effective against some groups of mammals but not others according to their body size (Wilson and Kerley, 2003). Using these arguments, we can infer from the architecture (shape, structure, vertical distribution of spines, timing of spine recruitment and pruning, association with defended organ) some information about the range of attack of herbivores, which organ is most likely defended, and which size class of animal is probably targeted by the defence.

In our analysis of trunk spine syndromes (Fig. 3; Supplementary Data Table S3), the most discriminating variables were the association in time and space between the spines and other organs (here specifically bark, leaf and reproductive organs), the mechanisms responsible for decreasing or increasing the spine density on the trunk through time, and specific characteristics associated with climbing plants. Apart from lianas, which had curved spines associated with non-self-supporting stems that are compatible with anchorage on neighbouring tree branches in a forested environment, the other syndromes probably have spines exclusively serving as a defence. Vertical distribution of spines suggests that the crown syndrome and the thorny trunk syndrome defend specifically against ground-dwelling mammals, while the prickly trunk syndrome could also defend against climbing animals (spines present at the base but extending high up onto the main branches). For the crown syndrome, our results corroborate the literature suggesting that their spines specifically defend soft edible parts such as leaves and axial meristems against medium to large ground-dwelling mammals (Belovsky *et al.*, 1991; Milewski and Madden, 2006). For the thorny trunk and prickly trunk syndromes, the asynchronous development of trunk spines with proximate leaves precludes this function. The localization and densities of their spines, and their ability to renew and reinforce them, rather suggest a protective function against debarking by medium to large animals for the thorny trunk spines and a protective function against debarking animals or/and climbing animals for the prickly trunk spines.

Finally, a caveat: although our clustering separated four clear morphological syndromes with different likely functions (climbing tools, shoot defence, trunk defence), the method does not allow us to evaluate function transfer during ontogeny: a species like *Gleditsia microphylla* that appears to establish spines associated with leaves in early development and with trunk in late development could defend its leaves and then its trunk. It would be important to conduct a detailed analysis of the ecological behaviour of each life stage for such species to understand better what is or are the function(s) of its spines. Additionally, species like *Sapium glandulosum*, which is weakly affiliated to any cluster due to a lack of species presenting this spiny strategy, question whether additional syndromes not revealed by our analysis remain to be found and advocate a more exhaustive morphological investigation of plants with spines on their primary stems.

### The challenge of testing trunk spine defensive function

In this study, we benefited from a unique opportunity provided by the living collection of the Xishuangbanna Tropical Botanical Garden to identify morphological strategies. On one hand, the living collection provided a large set of species (31 were investigated in this study) that could be described accurately and compared in similar conditions. On the other hand, because our measurements were made in a common garden environment, we did not observe the plants growing in their natural habitat subject to their natural herbivores; it is possible that our common garden measurements underestimate the spine densities that could be induced by herbivore attack (Milewski *et al.*, 1991). Our simulations (calibrated by real spine distribution) revealed strong differences in trunk spines on debarking and as obstacles for climbing among the plant morphological syndromes (Fig. 4; Supplementary Data Table S7). While simulations are not a formal demonstration of their effect nor an ecological demonstration of their function, they can provide indications about their potential function, the likely mammal size that might be affected by the defence, which characteristics are important for the defence, and how these differ according to the spine syndrome. The prickly trunk syndrome could slow down small to large climbing animals and defend the trunk against bark-feeding animals through the very high density of their spines, which restricts space for the mammals to insert their feet or mouths between spines. The thorny trunk syndrome was efficient against medium to large climbers and bark-feeding animals by producing large spines, branched or not, that are directionally complex, but was likely less effective than the prickly trunk syndrome because phyllotaxy ultimately constrained spine density.

Unfortunately, very little information is available in the literature to confirm our predictions about trunk spine effects on either debarking or climbing mammals. Species from both prickly trunk (*Bombax ceiba*, *Ceiba speciosa*, *Ceiba pentandra*) and thorny trunk syndromes (*Gleditsia microphylla*, *Flacourtia indica*) have been reported to have their bark targeted by mammals such as rodents, deer, elephants, cattle and primates (Bucher, 1987; Wenyuan *et al.*, 1993; Khan *et al.*, 1994; Steinheim *et al.*, 2005; Joshi and Singh, 2008; Jain *et al.*, 2011; Lapuente *et al.*, 2020), but no quantitative assessment of their effectiveness as defences has yet been attempted. Qualitative descriptions suggest that the establishment of spines on old saplings of *Bombax ceiba* (prickly trunk syndrome) and *Dalbergia sissoo* (thorny trunk syndrome) results in no further debarking by porcupines (Khan *et al.*, 2000), and make the debarking of *Ceiba pentandra* (prickly trunk syndrome) by chimpanzees more difficult (Lapuente *et al.*, 2020), which supports our predictions. These observations are particularly interesting as porcupines are highly specialized in debarking (Akram *et al.*, 2017), which suggests that trunk spines should induce an even stronger negative effect on less specialized herbivores. Evaluating this effect on less specialized mammalian herbivores that debark seasonally remains to be done but has a high importance for understanding the ecology of species with trunk spines, as non-specialist bark feeders likely dominate most herbivore-driven ecosystems. The effect of spines on climbing animals is even less documented, with only one study reporting trunk spines from *Ceiba pentandra* affecting the climbing of chimpanzees (Lapuente *et al.*, 2020). More investigations are necessary as effects on climbing herbivores are sometimes inconsistent: for example, spines of young rattan palms (*Calamus castaneus* and *Plectomia griffithii*) had no effect on the climbing rate of tree shrews (*Tupaia glis*) (Liu *et al.*, 2020), whereas spines of acacia trees (*Acacia rigidula*) were demonstrated to slow down the harvesting of food rewards by woodrats (*Neotoma micropus*) in the canopy (Cooper and Ginnett, 1998). Herbivore size, their sensitivity to spines, and the spine type are potentially important factors to evaluate in future experimental or observational work. Additionally, alternative functions of trunk spines should be included in further experimental work, such as their effect on rubbing and toppling by mammals.

Our simulation results would clearly need to be complemented by designed experiments to test the effects of trunk spine syndromes on animal performance, but we believe that our preliminary results provide some of the key factors that should be investigated (thorn versus cork spine, density and size). Moreover, a better understanding of plant defence syndromes could help to identify which herbivores were historically prominent in a landscape and be useful information for guiding rewilding efforts. In the specific case of trunk spines, their presence could suggest the historical effect of large debarking animals or climbing herbivores. For example, rewilding bark feeders could play a very important role in controlling canopy closure, thereby helping to maintain open habitat specialists (Savidge, 1968; Miquelle and Ballenberghe, 1989; Mountford, 1997; Akashi and Nakashizuka, 1999; Yokoyama *et al.*, 2001; Woods and Zeglen, 2003; Ihwagi *et al.*, 2009). Bark feeders might also favour adaptive traits for bark feeding other than spines, including: ability to regenerate bark (Wigley *et al.*, 2019); high bark thickness (Whitten and Whitten, 1987; Gill, 1992*a*; Månsson and Jarnemo, 2013), hardness and detachability (Whitten and Whitten, 1987; Gill, 1992*a*; Ando *et al.*, 2004; Klich, 2017); high branching rates (Anderson *et al.*, 1985; Gill, 1992*a*; Månsson and Jarnemo, 2013); or stem recruitment (O’Connor *et al.*, 2007).

### Do trunk spines defend attractive organs?

Several factors can make an organ more rewarding in terms of resource intake to animals: high nutrient concentration (e.g. bark with either high crude proteins or lower total phenol contents, or both), high available quantity (e.g. inner bark thickness), or nutrient value relative to other food sources available.

Comparisons among the identified syndromes showed that prickly trunk species have both a more nutritive bark and leaves than other syndromes and possess flowers attractive to mammals (Fig. 5; Supplementary Data Fig. S3, Supplementary Data Tables S2 and S13). For bark, the greater nutritiousness was not explained by nutrient concentration but by a greater thickness of the inner bark (twice as thick), which was also significantly thicker than that of confamilial non-spiny species, leading to a greater total nutrient resource. These observations are consistent with several reports suggesting that bark, leaves, flowers and fruits of prickly trunk trees are targeted by a large array of mammals (Bucher, 1987; Steinheim *et al.*, 2005; Vaughan *et al.*, 2007; Joshi and Singh, 2008; Jain *et al.*, 2011; Shyam and Saikia, 2012; Bessa *et al.*, 2015; Lapuente *et al.*, 2020). *Bombax ceiba* flowers could be targeted by mammals: they have been reported as part of the diet of up to 11 mammal species but mostly after falling on the ground (Jain *et al.*, 2011). Thorny trunk species are also reported as having their bark (Wenyuan *et al.*, 1993; Khan *et al.*, 1994) or flowers (Nishida and Uehara, 1983) attacked, but their bark nutritiousness and thickness did not differ either from the crown and liana syndromes or from their confamilial non-spiny species. This is at least partial support for the idea that that herbivores are choosing these species for their nutritiousness.

We expected spiny species to have more nutritious organs compared with their non-spiny confamilial species, but our results did not support this prediction (Fig. 5; Supplementary Data Fig. S3, Supplementary Data Table S2). It is possible that we were not measuring what is attractive to mammals; using a combination of nitrogen content and condensed tannins instead of total phenols might inform better about the available amount of crude protein in the bark. But a more plausible explanation is that the advantage provided by trunk spines to plant species is relative to their co-occurring species, rather than their allopatric confamilials, with which they rarely co-occur. In field conditions, a species that is nutritionally more attractive than its neighbours may require stronger defences against herbivory, hence the need for trunk spines. This can only be tested through in-field sampling in environments where spiny trunk species naturally occur, where nutrients can be measured in all community members and rates of bark herbivory measured. In some cases, it may not be possible to establish the advantages of spiny trunks conclusively, for several reasons. First, trunk spines have been suggested for several New World species (e.g. *Hura crepitans*, *Ceiba pentandra*, *Pachira quinata* and *Zanthoxylum setulosum*) as anachronistic defences against extinct megafauna present during the Pleistocene (Janzen and Martin, 1982), meaning that in some instances it will not be possible to assess their ecology *in situ*, as the major drivers of the defence might no longer be present in those systems. Second, in those areas where there are animal species that do feed on bark (e.g. the seasonal woodlands and forests of southeast Asia), animal densities are likely presently very low compared with their past densities, so it is unclear that the defensive utility can be demonstrated through community studies under present herbivory regimes.

### Conclusions

We present the first morphological investigation of trunk spine strategies across 31 tropical species, with a new suite of functional traits not yet referenced in trait databases or handbooks (e.g. Kattge *et al.*, 2012; Pérez-Harguindeguy *et al*., 2013), and investigated their potential functions using arguments from simulation and the nutritiousness of defended organs. Our results therefore provide an informed guide about trunk spine strategies and the criteria to identify them, their most likely function and the likely feeding mode and size of animal targeted by the defence, which could be used for setting up further experimental work. Our approach is not a demonstration of function *per se* but makes it possible (1) to qualify very contrasted morphological groups that could have emerged as adaptations to different constraints, (2) to identify the most likely functions, and (3) to refine the size range of animals that could be targeted when these are functioning as defences. We identified two morphological syndromes for which spines on the trunk are most probably not functional as a defence at the adult stage on the trunk and only present as a remnant of a function performed earlier in their ontogeny (anchoring the plant in lianas, or defending the canopy from ground mammals). We identified two new morphological syndromes that did not match previously identified functions of spines, that likely defend against debarking and climbing animals. Morphological, nutritional and simulation analyses make it possible to filter out which functions are unlikely for each strategy, but experimental work with bioassays and *in situ* observations would be required to draw conclusions about their ecological significance.

## SUPPLEMENTARY DATA

Supplementary data are available online at https://academic.oup.com/aob and consist of the following. Table S1: list of spiny and non-spiny species used in the study. Table S2: nutritiousness of leaf and inner bark for the 29 spiny species and 27 non-spiny species closely related phylogenetically. Table S3: morphological traits of the 31 spiny species. Table S4: variance explained by the first five eigenvectors made by the ordination. Table S5: coordinates of morphological variables for the first five eigenvectors made by the ordination. Table S6: coordinates of the 31 spiny species for the first five eigenvectors made by the ordination. Table S7: predicted damage by debarking and slowing mammal down estimated by computer simulations for the 31 spiny species following a size range of mouth and paw. Table S8: predictive posterior parameters and Bayesian goodness of fit for the Bayesian models of computer simulation analyses. Table S9: general non-linear hypothesis test of the Bayesian models for the computer simulation analyses. Table S10: Bayesian model selection for the nutritional analyses using the leave-one-out cross-validation information criterion method. Table S11: predictive posterior parameters and Bayesian goodness of fit for the Bayesian models of nutritional analyses. Table S12: general non-linear hypothesis test of the Bayesian models for the nutritional analyses. Table S13: predicted attractiveness of fruit and flower for the 31 spiny species. Figure S1: photographs of spine variables. Figure S2: simplified graphical representation of the computer simulation methodology about mammal debarking and climbing for spiny trunk species. Figure S3: median estimates of total phenol and nitrogen in leaves and inner bark, as well as inner bark thickness, for each spiny syndrome and for a non-spiny group with confamilial species.

mcac025_suppl_Supplementary-Material
