# Supplementary material for: Trunk spines of trees: a physical defence against bark removal and climbing by mammals?
Source: Ann Bot. 2022 Feb 24;129(5):541–54. doi: 10.1093/aob/mcac025 (PMC9007100; doi:10.1093/aob/mcac025)
Supplement: mcac025_suppl_Supplementary-Material [file mcac025_suppl_supplementary-material.doc]

Supporting information

**Table**

[Table S1: List of spiny and non-spiny species used in the study. 2](#__RefHeading___Toc95985313)

[Table S2: Nutritiousness of leaf and inner bark for the 29 spiny species and 27 non-spiny species closed phylogenetically. 3](#__RefHeading___Toc95985314)

[Table S3: Morphological traits of the 31 spiny species. 6](#__RefHeading___Toc95985315)

[Table S4: Variance explained by the first five eigenvectors made by the ordination. 8](#__RefHeading___Toc95985316)

[Table S5: Coordinates of morphological variables for the first five eigenvectors made by the ordination. 8](#__RefHeading___Toc95985317)

[Table S6: Coordinates of the 31 spiny species for the first five eigenvectors made by the ordination. 8](#__RefHeading___Toc95985318)

[Table S7: Predicted damage by debarking and slow mammal down estimated by computer simulations for the 31 spiny species following a range size of mouth and paw. 10](#__RefHeading___Toc95985319)

[Table S8: Predictive posterior parameters and Bayesian goodness-of-fit for the Bayesian models of computer simulation analyses. 15](#__RefHeading___Toc95985320)

[Table S9: General non-linear hypothesis test of the Bayesian models for the computer simulation analyses. 16](#__RefHeading___Toc95985321)

[Table S10: Bayesian model selection for the nutritional analyses using “Leave-One-Out cross-validation Information Criterion” method. 17](#__RefHeading___Toc95985322)

[Table S11: Predictive posterior parameters and Bayesian goodness-of-fit for the Bayesian models of nutritional analyses. 18](#__RefHeading___Toc95985323)

[Table S12: General non-linear hypothesis test of the Bayesian models for the nutritional analyses. 20](#__RefHeading___Toc95985324)

[Table S13: Predicted attractiveness of fruit and flower for the 31 spiny species. 22](#__RefHeading___Toc95985325)

**Figure**

[Figure S1: Simplified graphical representation of the computer simulation methodology about mammal debarking and climbing on spiny trunk species. 23](#__RefHeading___Toc95898404)

[Figure S2: Photographs of spine variables. 24](#__RefHeading___Toc95898405)

[Figure S3: Median estimates of total phenol and nitrogen in leaves and inner bark, as well as inner bark thickness, for each spiny syndrome and for a non-spiny group with confamilial species. 25](#__RefHeading___Toc95898406)

Table S1: List of spiny and non-spiny species used in the study.

| **N°** | **Family** | **Species** | **Spine** | **Native** |
| --- | --- | --- | --- | --- |
| 1 | Annonaceae | *Artabotrys hexapetalus* (L.f.) Bhandari | Yes | Asia |
|  |  | *Artabotrys siamensis* Miq. | No | Asia |
|  |  | *Fissistigma maclurei* Merr. | No | Asia |
|  |  | *Fissistigma tientangense* Tsiang & P.T.Li | No | Asia |
| 2 | Celastraceae | *Catha edulis* (Vahl) Endl. | No | Africa |
|  |  | *Maytenus austroyunnanensis* S.J. Pei & Y.H. Li | Yes | Asia |
|  |  | *Maytenus hookeri* Loes. | Yes | Asia |
| 3 | Combretaceae | *Combretum indicum* (L.) DeFilipps | Yes | Asia/Australia/Africa |
|  |  | *Combretum wallichii* var. *ternatum* (C.B.Clarke) Govaerts | No | Asia |
|  |  | *Getonia floribunda* Roxb. | No | Asia |
|  |  | *Quisqualis caudata* Craib | Yes | Asia |
|  |  | *Terminalia neotaliala* Capuron | No | Africa |
| 4 | Cornaceae | *Alangium chinense* (Lour.) Harms | No | Asia/Africa |
|  |  | *Alangium salviifolium* (L.f.) Wangerin | Yes | Asia/Africa |
| 5 | Elaeagnaceae | *Elaeagnus conferta* Roxb. | Yes | Asia |
| 6 | Euphorbiaceae | *Croton cascarilloides* Raeusch. | No | Asia |
|  |  | *Hura crepitans* L. | Yes | America |
|  |  | *Jatropha multifida* L. | No | America |
|  |  | *Sapium glandulosum* (L.) Morong | Yes | America |
| 7 | Fabaceae | *Acacia megaladena* Desv. | No | Asia |
|  |  | *Acacia pennata* (L.) Willd. | Yes | Asia |
|  |  | *Bauhinia variegata* var. *candida* Voigt | No | Asia |
|  |  | *Caesalpinia coriaria* (Jacq.) Wild. | No | America |
|  |  | *Caesalpinia cucullata* Roxb. | Yes | Asia |
|  |  | *Caesalpinia minax* Hance | Yes | Asia |
|  |  | *Cassia fistula* L. | No | Asia |
|  |  | *Cassia javanica subsp. agnes* (de Wit) K.Larsen | Yes | Asia |
|  |  | *Gleditsia microphylla* Isely | Yes | Asia |
|  |  | *Sophora velutina* Lindl. | No | Asia/Africa |
| 8 | Hypericaceae | *Cratoxylum cochinchinense* (Lour.) Blume | Yes | Asia |
| 9 | Lamiaceae | *Gmelina philippensis* Cham. | Yes | Asia |
|  |  | *Gmelina arborea* Roxb. | No | Asia |
|  |  | *Gmelina asiatica* L. | Yes | Asia |
|  |  | *Vitex glabrata* R.Br. | No | Asia/Australia |
|  |  | *Vitex negundo var. cannabifolia* (Siebold&Zucc.) Hand.-Mazz. | No | Asia |
| 10 | Malvaceae | *Bombax ceiba* L. | Yes | Asia/Australia |
|  |  | *Ceiba pentandra* (L.) Gaertn. | Yes | America |
|  |  | *Ceiba speciosa* (A.St.-Hil.) Ravenna | Yes | America |
|  |  | *Pachira aquatica* Aubl. | No | America |
|  |  | *Pachira quinata* (Jacq.) W.S.Alverson | Yes | America |
|  |  | *Sterculia nobilis Sm.* | No | Asia |
|  |  | *Theobroma cacao* L. | No | America |
| 11 | Moraceae | *Maclura cochinchinensis* (Lour.) Corner | Yes | Asia/Australia |
|  |  | *Streblus asper* Lour. | No | Asia |
| 12 | Olacaceae | *Olax scandens* Roxb. | Yes | Asia |
| 13 | Rhamnaceae | *Paliurus ramosissimus* (Lour.) Poir. | Yes | Asia |
|  |  | *Rhamnella wilsonii* C.K.Schneid. | Yes | Asia |
| 14 | Rubiaceae | *Catunaregam spinosa* (Thunb.) Tirveng. | Yes | Asia |
|  |  | *Duperrea pavettifolia* (Kurz) Pit. | No | Asia |
|  |  | *Gardenia tubifera* Wall. ex Roxb. | No | Asia |
|  |  | *Ixora cephalophora* Merr. | No | Asia |
|  |  | *Morinda angustifolia* Roxb. | No | Asia |
| 15 | Rutaceae | *Citrus lucida* (Scheff.) Mabb. | Yes | Asia |
|  |  | *Zanthoxylum armatum* DC. | Yes | Asia |
|  |  | *Zanthoxylum myriacanthum* Wall. Ex Hook. F. | Yes | Asia |
| 16 | Salicaceae | *Flacourtia indica* (Burm.f.) Merr. | Yes | Asia/Africa |
|  |  | *Flacourtia rukam* Zoll. & Moritzi | Yes | Asia |
|  |  | *Itoa orientalis* Hemsl. | No | Asia |

Table S2: Nutritiousness of leaf and inner bark for the 29 spiny species and 27 non-spiny species closed phylogenetically (N_Liana_= 7 sp., N_Crown_= 9 sp., N_Thorny_= 7 sp., N_Prickly_= 6 sp., N_Non-spiny_= 27 sp.).

|  |  |  |  | **Inner bark** | | | | | | **Leaf** | |
| --- | --- | --- | --- | --- | --- | --- | --- | --- | --- | --- | --- |
| **N°** | **Family** | **Species** | **Syndrome** | **N_march_ (mg/g)** | **N_may_ (mg/g)** | **P_march_ (%)** | **P_may_ (%)** | **Thickness_march_ (mm)** | **Thickness_may_ (mm)** | **N_may_ (mg/g)** | **P_may_ (%)** |
| 1 | Annonaceae | ***Artabotrys hexapetalus*** | Crown |  | 1.548 |  | 2.67 |  | 1 | 2.098 | 1.61 |
|  |  |  |  |  |  |  |  |  |  | 2.121 | 1.34 |
|  |  |  |  |  |  |  |  |  |  | 2.269 | 1.25 |
|  |  | *Artabotrys siamensis* | Non-spiny |  | 0.995 |  | 6.74 |  | 2 | 2.307 | 6.03 |
|  |  |  |  |  |  |  |  |  |  | 2.320 | 7.13 |
|  |  |  |  |  |  |  |  |  |  | 2.347 | 4.93 |
|  |  | *Fissistigma maclurei* | Non-spiny | 1.036 |  | 4.11 |  | 1 |  |  |  |
|  |  | *Fissistigma tientangense* | Non-spiny | 1.105 |  | 3.43 |  | 1 |  |  |  |
| 2 | Celastraceae | *Catha edulis* | Non-spiny |  |  |  |  |  |  | 1.637 | 7.54 |
|  |  | ***Maytenus austroyunnanensis*** | Crown | 1.009 | 0.987 | 5.11 | 5.19 | 1 | 1 | 1.821 | 5.26 |
|  |  |  |  |  |  |  |  |  |  | 1.843 | 4.35 |
|  |  |  |  |  |  |  |  |  |  | 2.019 | 3.77 |
|  |  | ***Maytenus hookeri*** | Crown | 0.878 | 0.889 | 4.66 | 4.49 | 1 | 1 | 1.200 | 7.25 |
| 3 | Combretaceae | ***Combretum indicum*** | Liana |  | 0.977 |  | 7.87 |  | 1 | 2.094 | 8.66 |
|  |  |  |  |  |  |  |  |  |  | 2.271 | 7.16 |
|  |  |  |  |  |  |  |  |  |  | 2.568 | 6.11 |
|  |  | *Combretum wallichii* var. *ternatum* | Non-spiny | 0.736 |  | 6.70 |  | 1 |  |  |  |
|  |  | *Getonia floribunda* | Non-spiny | 1.028 |  | 15.44 |  | 1 |  |  |  |
|  |  | ***Quisqualis caudata*** | Liana | 1.200 | 1.175 | 11.22 | 12.07 | 1 | 1 | 1.946 | 19.23 |
|  |  |  |  | 1.515 | 1.225 | 9.12 | 10.32 | 1 | 1 | 2.616 | 11.89 |
|  |  | *Terminalia neotaliala* | Non-spiny | 1.781 |  | 1.85 |  | 1 |  |  |  |
| 4 | Cornaceae | ***Alangium salviifolium*** | Thorny | 2.106 | 1.868 | 3.42 | 2.95 | 1 | 1 | 3.738 | 5.96 |
|  |  |  |  | 2.276 | 2.135 | 5.95 | 2.80 | 1 | 1 | 4.647 | 5.68 |
|  |  |  |  |  | 2.314 |  | 4.52 |  | 1 | 4.724 | 5.98 |
|  |  | *Alangium chinense* | Non-spiny |  |  |  |  |  |  | 3.653 | 2.10 |
| 5 | Elaeagnaceae | ***Elaeagnus conferta*** | Liana | 1.644 | 1.594 | 4.45 | 3.97 | 1 | 1 | 3.296 | 7.48 |
|  |  |  |  | 1.835 | 1.698 | 4.14 | 5.57 | 1 | 1 | 3.865 | 6.69 |
|  |  |  |  |  | 1.901 |  | 2.79 |  | 1 | 3.875 | 6.92 |
|  |  |  |  |  | 2.145 |  | 4.21 |  | 1 | 3.928 | 6.91 |
| 6 | Euphorbiaceae | *Croton cascarilloides* | Non-spiny |  | 2.302 |  | 2.06 | 2 |  | 2.840 | 2.36 |
|  |  |  |  |  |  |  |  |  |  | 3.379 | 3.17 |
|  |  | ***Hura crepitans*** | Prickly | 0.861 | 1.261 | 6.29 | 2.51 | 2 | 2 | 2.630 | 3.64 |
|  |  |  |  |  |  |  |  |  |  | 2.778 | 3.59 |
|  |  | *Jatropha multifida* | Non-spiny | 0.728 |  | 2.31 |  | 1 |  |  |  |
|  |  | ***Sapium glandulosum*** | Crown | 1.530 | 1.254 | 5.25 | 5.48 | 1 | 1 | 2.144 | 10.52 |
|  |  |  |  |  |  |  |  |  |  | 2.478 | 8.51 |
| 7 | Fabaceae | *Acacia megaladena* | Non-spiny | 2.413 |  | 1.23 |  | 1 |  | 3.131 | 4.40 |
|  |  | ***Acacia pennata*** | Liana |  | 1.516 |  | 8.33 | 1 |  |  |  |
|  |  | *Bauhinia variegata* var. *candida* | Non-spiny | 0.785 | 0.703 | 2.52 | 1.95 | 1 | 1 | 2.205 | 1.33 |
|  |  |  |  |  | 0.867 |  | 3.08 |  | 1 |  |  |
|  |  | *Caesalpinia coriaria* | Non-spiny | 1.878 | 1.519 | 7.04 | 7.19 | 1 | 2 | 2.323 | 13.67 |
|  |  |  |  |  |  |  |  |  |  | 2.812 | 9.16 |
|  |  | ***Caesalpinia minax*** | Liana | 0.926 | 1.760 | 0.44 | 0.99 | 1 | 1 | 3.473 | 2.73 |
|  |  | ***Caesalpinia cucullata*** | Liana |  | 0.927 |  | 3.43 | 1 |  | 1.439 | 6.47 |
|  |  |  |  |  |  |  |  |  |  | 1.465 | 5.48 |
|  |  |  |  |  |  |  |  |  |  | 1.729 | 4.09 |
|  |  | *Cassia fistula* | Non-spiny | 0.881 | 0.790 | 11.36 | 11.81 | 2 | 2 | 2.416 | 1.57 |
|  |  |  |  |  | 0.849 |  | 16.77 |  | 1 | 2.590 | 1.46 |
|  |  |  |  |  | 1.005 |  | 5.51 |  | 2 | 3.289 | 2.11 |
|  |  | ***Cassia javanica* subsp*. nodosa*** | Thorny | 1.298 | 1.126 | 7.20 | 6.70 | 1 | 1 | 2.139 | 5.54 |
|  |  |  |  | 1.442 | 1.270 | 9.25 | 8.69 | 1 | 1 | 2.718 | 3.69 |
|  |  |  |  |  | 1.499 |  | 6.20 |  | 1 | 2.934 | 4.04 |
|  |  | ***Gleditsia microphylla*** | Thorny | 0.679 | 0.921 | 5.43 | 3.45 | 1 | 1 | 1.509 | 7.82 |
|  |  |  |  |  |  |  |  |  |  | 1.797 | 5.95 |
|  |  |  |  |  |  |  |  |  |  | 2.004 | 6.26 |
|  |  | *Sophora velutina* | Non-spiny |  | 1.195 |  | 2.83 | 2 |  | 2.131 | 1.29 |
|  |  |  |  |  |  |  |  |  |  | 2.161 | 1.92 |
| 8 | Hypericaceae | ***Cratoxylum cochinchinense*** | Thorny |  | 1.102 |  | 9.89 | 1 |  | 2.195 | 3.82 |
| 9 | Lamiaceae | *Gmelina arborea* | Non-spiny |  |  |  |  |  |  | 0.717 | 2.51 |
|  |  |  |  |  |  |  |  |  |  | 2.128 | 1.99 |
|  |  | ***Gmelina asiatica*** | Crown | 1.170 | 1.661 | 4.62 | 4.28 | 1 | 1 | 2.133 | 6.39 |
|  |  |  |  |  |  |  |  |  |  | 2.219 | 3.37 |
|  |  |  |  |  |  |  |  |  |  | 2.625 | 3.67 |
|  |  | ***Gmelina philippensis*** | Crown | 0.910 | 0.737 | 2.09 | 2.07 | 1 | 1 | 1.839 | 1.53 |
|  |  |  |  |  |  |  |  |  |  | 2.069 | 1.32 |
|  |  |  |  |  |  |  |  |  |  | 2.148 | 1.33 |
|  |  | *Vitex glarata* | Non-spiny | 1.048 |  | 6.71 |  | 1 |  |  |  |
|  |  | *Vitex negundo* var. *cannabifolia* | Non-spiny | 1.187 |  | 6.23 |  | 1 |  |  |  |
| 10 | Malvaceae | ***Bombax ceiba*** | Prickly | 0.466 |  | 2.11 |  | 3 |  | 1.358 | 3.29 |
|  |  |  |  |  |  |  |  |  |  | 1.538 | 5.13 |
|  |  | ***Ceiba pentandra*** | Prickly | 0.883 | 0.554 | 3.04 | 2.54 | 3 | 2 | 1.864 | 0.48 |
|  |  |  |  |  | 1.008 |  | 5.70 |  | 3 | 2.041 | 0.51 |
|  |  |  |  |  | 1.086 |  | 0.89 |  | 3 |  |  |
|  |  | ***Ceiba speciosa*** | Prickly | 0.622 | 0.896 | 1.87 | 4.20 | 4 | 4 | 2.071 | 0.51 |
|  |  |  |  | 1.209 | 1.522 | 2.80 | 1.39 | 5 | 5 | 2.341 | 0.61 |
|  |  |  |  |  |  |  |  |  |  | 2.673 | 0.62 |
|  |  |  |  |  |  |  |  |  |  | 2.713 | 0.81 |
|  |  |  |  |  |  |  |  |  |  | 2.873 | 0.78 |
|  |  | *Pachira aquatica* | Non-spiny | 1.590 | 1.337 | 2.41 | 1.28 | 2 | 3 | 2.059 | 1.37 |
|  |  |  |  | 1.741 | 1.383 | 0.71 | 0.83 | 3 | 3 | 2.077 | 2.60 |
|  |  |  |  |  |  |  |  |  |  | 2.240 | 1.35 |
|  |  |  |  |  |  |  |  |  |  | 2.288 | 1.68 |
|  |  |  |  |  |  |  |  |  |  | 2.297 | 1.18 |
|  |  |  |  |  |  |  |  |  |  | 2.535 | 1.70 |
|  |  | ***Pachira quinata*** | Prickly | 0.755 | 0.656 | 4.73 | 3.81 | 2 | 2 | 2.334 | 2.06 |
|  |  |  |  |  |  |  |  |  |  | 2.497 | 1.54 |
|  |  | *Sterculia nobilis* | Non-spiny | 0.852 |  | 5.07 |  | 1 |  |  |  |
|  |  | *Theobroma cacao* | Non-spiny | 1.023 | 1.013 | 4.34 | 6.42 | 1 | 1 | 1.137 | 2.80 |
|  |  |  |  |  | 1.032 |  | 2.26 |  | 1 | 1.198 | 4.35 |
|  |  |  |  |  |  |  |  |  |  | 1.680 | 2.10 |
| 11 | Moraceae | ***Maclura cochinchinensis*** | Liana | 1.122 | 1.288 | 0.46 | 3.16 | 1 | 1 | 2.981 | 2.28 |
|  |  |  |  |  |  |  |  |  |  | 3.038 | 1.35 |
|  |  | *Streblus asper* | Non-spiny | 0.973 | 0.898 | 5.43 | 6.69 | 2 | 2 | 0.984 | 1.49 |
|  |  |  |  |  | 1.048 |  | 4.16 |  | 2 | 1.765 | 2.46 |
|  |  |  |  |  |  |  |  |  |  | 2.145 | 1.56 |
| 12 | Rhamnaceae | ***Paliurus ramosissimus*** | Crown | 1.246 |  | 3.53 |  | 1 |  |  |  |
|  |  | ***Rhamnella wilsonii*** | Crown |  | 0.921 |  | 1.86 | 2 |  | 1.319 | 1.42 |
|  |  |  |  |  |  |  |  |  |  | 1.614 | 4.36 |
| 13 | Rubiaceae | ***Catunaregam spinosa*** | Crown | 1.168 | 1.094 | 2.39 | 2.49 | 1 | 1 | 2.161 | 1.90 |
|  |  |  |  | 1.533 | 1.197 | 1.73 | 2.09 | 1 | 1 | 2.213 | 1.34 |
|  |  |  |  |  | 1.213 |  | 2.60 |  | 1 | 2.226 | 1.88 |
|  |  | *Duperrea pavettifolia* | Non-spiny | 1.423 |  | 13.54 |  | 1 |  |  |  |
|  |  | *Gardenia tubifera* | Non-spiny |  | 0.904 |  | 4.74 | 1 |  | 1.215 | 5.08 |
|  |  |  |  |  |  |  |  |  |  | 1.320 | 4.02 |
|  |  | *Ixora cephalophora* | Non-spiny | 1.142 |  | 5.90 |  | 1 |  |  |  |
|  |  | *Morinda angustifolia* | Non-spiny | 0.990 |  | 0.39 |  | 1 |  |  |  |
| 14 | Rutaceae | ***Citrus lucida*** | Thorny | 0.850 | 0.801 | 0.57 | 1.34 | 1 | 2 | 2.078 | 3.82 |
|  |  |  |  |  |  |  |  |  |  | 2.189 | 3.56 |
|  |  | ***Zanthoxylum armatum*** | Prickly | 2.635 | 1.023 | 4.91 | 1.49 | 2 | 2 | 0.744 | 0.44 |
|  |  |  |  |  |  |  |  |  |  | 3.601 | 3.57 |
|  |  |  |  |  |  |  |  |  |  | 2.992 | 1.42 |
| 15 | Salicaceae | ***Flacourtia indica*** | Thorny | 1.089 | 1.009 | 5.32 | 3.46 | 1 | 1 | 1.647 | 4.38 |
|  |  |  |  |  |  |  |  |  |  | 2.484 | 3.00 |
|  |  |  |  |  |  |  |  |  |  | 2.500 | 3.58 |
|  |  | ***Flacourtia rukam*** | Thorny | 0.804 |  | 4.55 |  | 1 |  |  |  |
|  |  | *Itoa orientalis* | Non-spiny |  | 1.987 |  | 3.74 | 2 |  | 1.582 | 2.67 |

Table S3: Morphological traits of the 31 spiny species.

| **Species** | **Liana** | **Curved** | **Straightened** | **Branched** | **Mixed** | **Phyllotaxic** | **Extended** | **Immediate** | **Renewal** | **Maintained** | **Length** | **Density** | **Origin** | **Syndrome** |
| --- | --- | --- | --- | --- | --- | --- | --- | --- | --- | --- | --- | --- | --- | --- |
| Artabotrys hexapetalus | 0 | 0 | 0 | 0 | 0 | 1 | 0 | 1 | 0 | 1 | 2.5 | 0.058 | Thorn | Crown |
| Catunaregam spinosa | 0 | 0 | 0 | 0 | 0 | 1 | 0 | 1 | 0 | 0 | 2.3 | 0.083 | Thorn | Crown |
| Gmelina asiatica | 0 | 0 | 0 | 0 | 0 | 1 | 0 | 1 | 0 | 0 | 2.5 | 0.117 | Thorn | Crown |
| Gmelina philippensis | 1 | 0 | 0 | 0 | 0 | 1 | 0 | 1 | 0 | 0 | 2.5 | 0.058 | Thorn | Crown |
| Maytenus austroyunnanensis | 1 | 0 | 0 | 0 | 0 | 1 | 0 | 1 | 0 | 0 | 3.0 | 0.083 | Thorn | Crown |
| Maytenus hookeri | 0 | 0 | 0 | 0 | 0 | 1 | 0 | 1 | 0 | 0 | 1.0 | 0.075 | Thorn | Crown |
| Paliurus ramosissimus | 0 | 0 | 0 | 0 | 0 | 1 | 0 | 1 | 0 | 0 | 1.0 | 0.158 | Stipule | Crown |
| Rhamnella wilsonii | 0 | 0 | 0 | 0 | 0 | 1 | 0 | 1 | 0 | 0 | 3.0 | 0.044 | Thorn | Crown |
| Sapium glandulosum | 0 | 0 | 0 | 0 | 0 | 1 | 0 | 0 | 0 | 1 | 6.5 | 0.027 | Thorn | Crown |
| Acacia pennate | 1 | 1 | 0 | 0 | 0 | 0 | 1 | 1 | 0 | 0 | 0.3 | 0.097 | Prickle | Liana |
| Caesalpinia cucullata | 1 | 1 | 0 | 0 | 0 | 0 | 1 | 1 | 0 | 1 | 1.5 | 0.108 | Prickle | Liana |
| Caesalpinia minax | 1 | 0 | 0 | 0 | 0 | 0 | 1 | 1 | 0 | 1 | 0.3 | 0.450 | Prickle | Liana |
| Combretum indicum | 1 | 1 | 0 | 0 | 0 | 1 | 1 | 0 | 0 | 0 | 2.5 | 0.033 | Leaf | Liana |
| Elaeagnus conferta | 1 | 1 | 1 | 0 | 1 | 1 | 0 | 1 | 1 | 1 | 3.0 | 0.038 | Thorn | Liana |
| Maclura cochinchinensis | 1 | 1 | 1 | 0 | 0 | 1 | 1 | 1 | 0 | 1 | 2.0 | 0.083 | Thorn | Liana |
| Olax scandens | 1 | 1 | 0 | 0 | 0 | 1 | 1 | 0 | 0 | 1 | 5.0 | 0.025 | Thorn | Liana |
| Quisqualis cuadata | 1 | 1 | 0 | 0 | 0 | 1 | 1 | 0 | 0 | 0 | 2.5 | 0.100 | Leaf | Liana |
| Bombax ceiba | 0 | 0 | 0 | 0 | 0 | 0 | 0 | 0 | 1 | 1 | 2.0 | 0.183 | Cork | Prickly |
| Ceiba pentandra | 0 | 0 | 0 | 0 | 0 | 0 | 1 | 0 | 1 | 1 | 1.5 | 0.178 | Cork | Prickly |
| Ceiba speciosa | 0 | 0 | 0 | 0 | 0 | 0 | 1 | 0 | 1 | 1 | 3.0 | 0.180 | Cork | Prickly |
| Hura crepitans | 0 | 0 | 0 | 0 | 0 | 0 | 1 | 0 | 1 | 1 | 1.2 | 0.417 | Cork | Prickly |
| Pachira quinata | 0 | 0 | 0 | 0 | 0 | 0 | 1 | 0 | 1 | 1 | 2.0 | 0.428 | Cork | Prickly |
| Zanthoxylum armatum | 0 | 0 | 0 | 0 | 0 | 0 | 1 | 1 | 1 | 1 | 2.5 | 0.192 | Prickle/ Cork | Prickly |
| Zanthoxylum myriacanthum | 0 | 0 | 0 | 0 | 0 | 0 | 1 | 1 | 1 | 1 | 3.0 | 0.142 | Prickle/ Cork | Prickly |
| Alangium salviifolium | 0 | 0 | 0 | 0 | 1 | 1 | 0 | 1 | 1 | 1 | 12.0 | 0.083 | Thorn | Thorny |
| Cassia javanica | 0 | 0 | 0 | 0 | 1 | 1 | 1 | 0 | 1 | 1 | 7.0 | 0.065 | Thorn | Thorny |
| Citrus lucida | 0 | 0 | 0 | 1 | 1 | 1 | 0 | 1 | 1 | 1 | 5.0 | 0.133 | Thorn | Thorny |
| Cratoxylum cochinchinense | 0 | 0 | 0 | 0 | 1 | 1 | 0 | 0 | 1 | 1 | 6.0 | 0.080 | Thorn | Thorny |
| Flacourtia indica | 0 | 0 | 0 | 1 | 1 | 1 | 0 | 1 | 1 | 0 | 8.0 | 0.371 | Thorn | Thorny |
| Flacourtia rukam | 0 | 0 | 0 | 1 | 1 | 1 | 0 | 1 | 1 | 1 | 7.0 | 0.094 | Thorn | Thorny |
| Gleditsia microphylla | 0 | 0 | 0 | 1 | 1 | 1 | 0 | 1 | 1 | 0 | 23.0 | 0.300 | Thorn | Thorny |

Table S4: Variance explained by the first five eigenvectors made by the ordination.

|  |  |  | **Axe 1** | **Axe 2** | **Axe 3** | **Axe 4** | **Axe 5** |
| --- | --- | --- | --- | --- | --- | --- | --- |
|  |  | **Eigenvalues** | 3.596 | 2.790 | 1.883 | 1.324 | 0.894 |
|  | **Projected inertia (%)** | | 29.969 | 23.251 | 15.692 | 11.032 | 7.453 |
| **Cumulative projected inertia (%)** | | | 29.970 | 53.220 | 68.910 | 79.940 | 87.400 |

Table S5: Coordinates of morphological variables for the first five eigenvectors made by the ordination.

| **Variables** | **CS1** | **CS2** | **CS3** | **CS4** | **CS5** |
| --- | --- | --- | --- | --- | --- |
| **Non-self-supporting stem** | 0.37609534 | 0.22186618 | 0.21383454 | -0.22540902 | 0.25754655 |
| **High spine density** | -0.12334906 | -0.40999741 | -0.17516910 | -0.45881463 | 0.15479961 |
| **Long spine** | -0.39070469 | 0.15696971 | 0.24174534 | 0.26925070 | 0.17584139 |
| **Curved spine** | 0.34056895 | 0.19812149 | 0.39108326 | -0.12575591 | 0.30700640 |
| **Straightened spine** | 0.10707708 | 0.19026495 | 0.46109273 | -0.30520433 | -0.46528360 |
| **Branched spine** | -0.38102113 | 0.07998315 | 0.04765585 | -0.35499674 | 0.42310069 |
| **Mixed spine** | -0.40604424 | 0.11683061 | 0.32852160 | -0.13903748 | 0.11436299 |
| **Phyllotaxic spine** | -0.17008173 | 0.52045474 | 0.02423535 | 0.19616223 | 0.03186497 |
| **Immediate spine** | -0.07058874 | 0.25965400 | -0.22174384 | -0.59637140 | -0.32196825 |
| **Maintained spine** | -0.04478329 | -0.32269361 | 0.44084924 | 0.10771090 | -0.40384954 |
| **Spine renewal** | -0.34607640 | -0.32954706 | 0.29997157 | -0.06996565 | -0.01701608 |
| **Extended spine production** | 0.31225059 | -0.33393516 | 0.23518278 | -0.03507615 | 0.32846444 |

Table S6: Coordinates of the 31 spiny species for the first five eigenvectors made by the ordination.

| **Species** | **Axis1** | **Axis2** | **Axis3** | **Axis4** | **Axis5** |
| --- | --- | --- | --- | --- | --- |
| **Maclura cochinchinensis** | 2.615750617 | 1.640502888 | 2.724590006 | -1.636318510 | -1.329714580 |
| **Caesalpinia cucullata** | 2.626172119 | -0.436468424 | 0.659478373 | -1.055054370 | 0.492660780 |
| **Caesalpinia minax** | 2.280134238 | -1.944210434 | -1.028548805 | -2.077881120 | -0.269758380 |
| **Acacia pennata** | 3.430686578 | 0.003400108 | -0.651660417 | -1.691925070 | 0.987319560 |
| **Combretum indicum** | 2.469638548 | 1.512196989 | 0.674079186 | 1.243431290 | 1.925550020 |
| **Quisqualis caudata** | 2.293303114 | 0.926079274 | 0.423663670 | 0.587526210 | 2.146846030 |
| **Olax scandens** | 2.122325032 | 1.116814168 | 1.827194848 | 1.835235150 | 1.175826960 |
| **Elaeagnus conferta** | 0.316884304 | 2.402504891 | 3.887924304 | -1.440637320 | -1.839517900 |
| **Rhamnella wilsonii** | -0.048966578 | 1.656998651 | -1.670974155 | 0.734550110 | -0.599726870 |
| **Gmelina asiatica** | -0.125730541 | 1.108313357 | -1.940621808 | 0.101661510 | -0.439974010 |
| **Maytenus austroyunnanensis** | 0.654635041 | 1.796093979 | -1.356888313 | -0.123116710 | 0.077897860 |
| **Maytenus hookeri** | 0.340955306 | 1.184325744 | -2.085175340 | 0.091875140 | -0.706941580 |
| **Gmelina philippensis** | 0.790425806 | 1.953914962 | -1.324685109 | 0.034622650 | -0.029099490 |
| **Paliurus ramosissimus** | 0.222444274 | 0.790409367 | -2.253473909 | -0.348943730 | -0.558213570 |
| **Catunaregam spinosa** | -0.035090790 | 1.275347570 | -1.885366886 | 0.279952810 | -0.524722030 |
| **Artabotrys hexapetalus** | -0.106059779 | 0.816800014 | -0.877046062 | 0.737951550 | -1.409157180 |
| **Sapium glandulosum** | -0.249821478 | 0.844232428 | 0.014588006 | 2.721284940 | -0.703024060 |
| **Flacourtia indica** | -3.568958375 | 0.546299869 | -0.397085052 | -1.751397680 | 1.506002450 |
| **Flacourtia rukam** | -3.384832377 | 0.586435162 | 0.782387158 | -0.757792980 | 0.376878320 |
| **Gleditsia microphylla** | -3.991525489 | 0.841944522 | -0.066740811 | -1.311234750 | 1.668989360 |
| **Citrus lucida** | -3.294632159 | 0.344541623 | 0.614032314 | -1.063317830 | 0.380713490 |
| **Cassia javanica** | -1.414601690 | -0.670823798 | 1.659589315 | 1.695351130 | 0.374089470 |
| **Alangium salviifolium** | -2.461377646 | 0.507219482 | 0.812457072 | 0.535296000 | -0.805235730 |
| **Cratoxylum cochinchinense** | -2.008460147 | -0.136339431 | 1.098889349 | 1.597085060 | -0.274471340 |
| **Zanthoxylum armatum** | 0.002318575 | -2.259862405 | -0.126431433 | -0.600364660 | -0.612406730 |
| **Zanthoxylum myriacanthum** | -0.028487132 | -2.068725720 | -0.009544424 | -0.367596960 | -0.637162670 |
| **Hura crepitans** | 0.343661593 | -3.340003374 | -0.034413298 | -0.031365900 | 0.072579460 |
| **Pachira quinata** | 0.118776832 | -3.265082339 | 0.096288937 | 0.105353600 | 0.177124910 |
| **Ceiba speciosa** | 0.081328666 | -2.736771303 | 0.400343631 | 0.738540620 | 0.083089860 |
| **Bombax ceiba** | -0.373532558 | -2.144884078 | -0.184385789 | 0.678497530 | -0.652494780 |
| **Ceiba pentandra** | 0.382636097 | -2.851203742 | 0.217535440 | 0.538732290 | -0.053947650 |

Table S7: Predicted damage by debarking and slow mammal down estimated by computer simulations for the 31 spiny species following a range size of mouth and paw.

| **Syndrome** | **Species** | **Proportion of bark (%)** | **Probability of ring (%)** | **Slowing down** | **Size** |
| --- | --- | --- | --- | --- | --- |
| Crown | Artabortys hexapetalus | 85.9 | 68.7 | 23.3 | 1 |
| Crown | Artabortys hexapetalus | 66.6 | 54.0 | 133.3 | 2 |
| Crown | Artabortys hexapetalus | 55.7 | 40.7 | 570.0 | 3 |
| Crown | Artabortys hexapetalus | 44.8 | 27.3 | 954.5 | 4 |
| Crown | Artabortys hexapetalus | 34.5 | 16.0 | 954.5 | 5 |
| Crown | Artabortys hexapetalus | 24.7 | 6.7 | 954.5 | 6 |
| Crown | Artabortys hexapetalus | 16.9 | 1.3 | 954.5 | 7 |
| Crown | Artabortys hexapetalus | 12.7 | 0.0 | 954.5 | 8 |
| Crown | Artabortys hexapetalus | 10.6 | 0.0 | 954.5 | 9 |
| Crown | Artabortys hexapetalus | 8.4 | 0.0 | 954.5 | 10 |
| Crown | Catunaregan spinosa | 78.9 | 68.7 | 66.7 | 1 |
| Crown | Catunaregan spinosa | 65.7 | 52.0 | 333.3 | 2 |
| Crown | Catunaregan spinosa | 53.1 | 36.7 | 333.3 | 3 |
| Crown | Catunaregan spinosa | 41.8 | 23.3 | 333.3 | 4 |
| Crown | Catunaregan spinosa | 30.8 | 10.0 | 333.3 | 5 |
| Crown | Catunaregan spinosa | 22.4 | 3.7 | 333.3 | 6 |
| Crown | Catunaregan spinosa | 17.2 | 0.3 | 333.3 | 7 |
| Crown | Catunaregan spinosa | 13.3 | 0.0 | 333.3 | 8 |
| Crown | Catunaregan spinosa | 10.1 | 0.0 | 333.3 | 9 |
| Crown | Catunaregan spinosa | 7.7 | 0.0 | 333.3 | 10 |
| Crown | Gmelina asiatica | 70.5 | 36.0 | 46.7 | 1 |
| Crown | Gmelina asiatica | 52.6 | 14.0 | 446.7 | 2 |
| Crown | Gmelina asiatica | 36.7 | 1.3 | 446.7 | 3 |
| Crown | Gmelina asiatica | 24.6 | 0.0 | 446.7 | 4 |
| Crown | Gmelina asiatica | 16.7 | 0.0 | 446.7 | 5 |
| Crown | Gmelina asiatica | 11.4 | 0.0 | 446.7 | 6 |
| Crown | Gmelina asiatica | 7.3 | 0.0 | 446.7 | 7 |
| Crown | Gmelina asiatica | 4.1 | 0.0 | 446.7 | 8 |
| Crown | Gmelina asiatica | 2.5 | 0.0 | 446.7 | 9 |
| Crown | Gmelina asiatica | 1.2 | 0.0 | 446.7 | 10 |
| Crown | Gmelina phyllipensis | 84.0 | 63.7 | 33.3 | 1 |
| Crown | Gmelina phyllipensis | 74.6 | 43.7 | 85.0 | 2 |
| Crown | Gmelina phyllipensis | 65.1 | 25.7 | 85.0 | 3 |
| Crown | Gmelina phyllipensis | 56.0 | 10.7 | 85.0 | 4 |
| Crown | Gmelina phyllipensis | 47.2 | 0.7 | 85.0 | 5 |
| Crown | Gmelina phyllipensis | 38.9 | 0.0 | 85.0 | 6 |
| Crown | Gmelina phyllipensis | 30.9 | 0.0 | 85.0 | 7 |
| Crown | Gmelina phyllipensis | 23.1 | 0.0 | 85.0 | 8 |
| Crown | Gmelina phyllipensis | 17.2 | 0.0 | 85.0 | 9 |
| Crown | Gmelina phyllipensis | 12.5 | 0.0 | 85.0 | 10 |
| Crown | Maytenus austroyannensis | 79.4 | 51.7 | 23.3 | 1 |
| Crown | Maytenus austroyannensis | 68.2 | 33.0 | 246.7 | 2 |
| Crown | Maytenus austroyannensis | 57.6 | 20.3 | 246.7 | 3 |
| Crown | Maytenus austroyannensis | 48.3 | 13.3 | 246.7 | 4 |
| Crown | Maytenus austroyannensis | 40.0 | 7.7 | 246.7 | 5 |
| Crown | Maytenus austroyannensis | 32.6 | 4.3 | 246.7 | 6 |
| Crown | Maytenus austroyannensis | 26.4 | 1.0 | 246.7 | 7 |
| Crown | Maytenus austroyannensis | 21.3 | 0.0 | 246.7 | 8 |
| Crown | Maytenus austroyannensis | 17.4 | 0.0 | 246.7 | 9 |
| Crown | Maytenus austroyannensis | 14.4 | 0.0 | 246.7 | 10 |
| Crown | Maytenus hookeri | 79.2 | 52.0 | 56.7 | 1 |
| Crown | Maytenus hookeri | 66.5 | 25.3 | 155.0 | 2 |
| Crown | Maytenus hookeri | 54.9 | 10.0 | 155.0 | 3 |
| Crown | Maytenus hookeri | 43.9 | 2.7 | 155.0 | 4 |
| Crown | Maytenus hookeri | 34.5 | 0.0 | 146.7 | 5 |
| Crown | Maytenus hookeri | 27.4 | 0.0 | 145.0 | 6 |
| Crown | Maytenus hookeri | 21.1 | 0.0 | 155.0 | 7 |
| Crown | Maytenus hookeri | 16.0 | 0.0 | 143.3 | 8 |
| Crown | Maytenus hookeri | 12.9 | 0.0 | 155.0 | 9 |
| Crown | Maytenus hookeri | 10.0 | 0.0 | 155.0 | 10 |
| Crown | Paliurus ramossissimus | 67.7 | 44.7 | 78.9 | 1 |
| Crown | Paliurus ramossissimus | 49.5 | 20.3 | 466.7 | 2 |
| Crown | Paliurus ramossissimus | 35.3 | 8.3 | 466.7 | 3 |
| Crown | Paliurus ramossissimus | 24.5 | 2.7 | 466.7 | 4 |
| Crown | Paliurus ramossissimus | 15.6 | 0.0 | 466.7 | 5 |
| Crown | Paliurus ramossissimus | 10.8 | 0.0 | 466.7 | 6 |
| Crown | Paliurus ramossissimus | 7.3 | 0.0 | 466.7 | 7 |
| Crown | Paliurus ramossissimus | 4.2 | 0.0 | 466.7 | 8 |
| Crown | Paliurus ramossissimus | 2.6 | 0.0 | 466.7 | 9 |
| Crown | Paliurus ramossissimus | 1.5 | 0.0 | 466.7 | 10 |
| Crown | Rhamna willsoni | 86.7 | 33.3 | 0.0 | 1 |
| Crown | Rhamna willsoni | 67.2 | 12.0 | 46.7 | 2 |
| Crown | Rhamna willsoni | 49.4 | 4.0 | 300.0 | 3 |
| Crown | Rhamna willsoni | 41.0 | 0.0 | 545.5 | 4 |
| Crown | Rhamna willsoni | 33.9 | 0.0 | 1166.7 | 5 |
| Crown | Rhamna willsoni | 27.8 | 0.0 | 1166.7 | 6 |
| Crown | Rhamna willsoni | 24.1 | 0.0 | 1166.7 | 7 |
| Crown | Rhamna willsoni | 21.3 | 0.0 | 1166.7 | 8 |
| Crown | Rhamna willsoni | 18.7 | 0.0 | 1166.7 | 9 |
| Crown | Rhamna willsoni | 16.5 | 0.0 | 1166.7 | 10 |
| Crown | Sapium glandulosum | 91.5 | 78.7 | 0.0 | 1 |
| Crown | Sapium glandulosum | 86.1 | 65.3 | 66.7 | 2 |
| Crown | Sapium glandulosum | 80.8 | 52.0 | 66.7 | 3 |
| Crown | Sapium glandulosum | 75.5 | 38.7 | 66.7 | 4 |
| Crown | Sapium glandulosum | 70.3 | 27.7 | 66.7 | 5 |
| Crown | Sapium glandulosum | 65.1 | 18.0 | 66.7 | 6 |
| Crown | Sapium glandulosum | 60.4 | 11.3 | 66.7 | 7 |
| Crown | Sapium glandulosum | 55.6 | 4.7 | 66.7 | 8 |
| Crown | Sapium glandulosum | 51.0 | 0.0 | 66.7 | 9 |
| Crown | Sapium glandulosum | 47.7 | 0.0 | 66.7 | 10 |
| Liana | Acacia pennata | 75.4 | 27.0 | 0.0 | 1 |
| Liana | Acacia pennata | 42.0 | 10.3 | 266.7 | 2 |
| Liana | Acacia pennata | 19.8 | 5.7 | 700.0 | 3 |
| Liana | Acacia pennata | 12.1 | 2.3 | 1636.4 | 4 |
| Liana | Acacia pennata | 8.1 | 0.0 | 2166.7 | 5 |
| Liana | Acacia pennata | 5.6 | 0.0 | 2166.7 | 6 |
| Liana | Acacia pennata | 4.2 | 0.0 | 2166.7 | 7 |
| Liana | Acacia pennata | 2.8 | 0.0 | 2166.7 | 8 |
| Liana | Acacia pennata | 2.1 | 0.0 | 2166.7 | 9 |
| Liana | Acacia pennata | 1.7 | 0.0 | 2166.7 | 10 |
| Liana | Caesalpinia cucullata | 73.0 | 40.7 | 46.7 | 1 |
| Liana | Caesalpinia cucullata | 58.0 | 20.3 | 357.8 | 2 |
| Liana | Caesalpinia cucullata | 44.0 | 6.7 | 357.8 | 3 |
| Liana | Caesalpinia cucullata | 31.1 | 0.0 | 357.8 | 4 |
| Liana | Caesalpinia cucullata | 18.9 | 0.0 | 357.8 | 5 |
| Liana | Caesalpinia cucullata | 9.8 | 0.0 | 357.8 | 6 |
| Liana | Caesalpinia cucullata | 4.5 | 0.0 | 357.8 | 7 |
| Liana | Caesalpinia cucullata | 2.4 | 0.0 | 357.8 | 8 |
| Liana | Caesalpinia cucullata | 1.6 | 0.0 | 357.8 | 9 |
| Liana | Caesalpinia cucullata | 1.1 | 0.0 | 357.8 | 10 |
| Liana | Caesalpinia minax | 25.8 | 15.3 | 305.6 | 1 |
| Liana | Caesalpinia minax | 7.6 | 1.7 | 1600.0 | 2 |
| Liana | Caesalpinia minax | 2.3 | 0.0 | 1600.0 | 3 |
| Liana | Caesalpinia minax | 0.5 | 0.0 | 1600.0 | 4 |
| Liana | Caesalpinia minax | 0.1 | 0.0 | 1600.0 | 5 |
| Liana | Caesalpinia minax | 0.0 | 0.0 | 1600.0 | 6 |
| Liana | Caesalpinia minax | 0.0 | 0.0 | 1600.0 | 7 |
| Liana | Caesalpinia minax | 0.0 | 0.0 | 1600.0 | 8 |
| Liana | Caesalpinia minax | 0.0 | 0.0 | 1600.0 | 9 |
| Liana | Caesalpinia minax | 0.0 | 0.0 | 1600.0 | 10 |
| Liana | Combretum indicum | 87.2 | 68.0 | 14.4 | 1 |
| Liana | Combretum indicum | 79.2 | 48.0 | 133.3 | 2 |
| Liana | Combretum indicum | 71.3 | 29.3 | 133.3 | 3 |
| Liana | Combretum indicum | 63.4 | 12.7 | 133.3 | 4 |
| Liana | Combretum indicum | 56.4 | 4.0 | 133.3 | 5 |
| Liana | Combretum indicum | 50.0 | 0.0 | 133.3 | 6 |
| Liana | Combretum indicum | 44.0 | 0.0 | 133.3 | 7 |
| Liana | Combretum indicum | 38.4 | 0.0 | 133.3 | 8 |
| Liana | Combretum indicum | 33.2 | 0.0 | 133.3 | 9 |
| Liana | Combretum indicum | 28.3 | 0.0 | 133.3 | 10 |
| Liana | Elaeagnus conferta | 88.3 | 64.7 | 13.3 | 1 |
| Liana | Elaeagnus conferta | 75.2 | 44.7 | 113.3 | 2 |
| Liana | Elaeagnus conferta | 65.7 | 25.0 | 300.0 | 3 |
| Liana | Elaeagnus conferta | 56.6 | 12.0 | 300.0 | 4 |
| Liana | Elaeagnus conferta | 48.3 | 4.0 | 300.0 | 5 |
| Liana | Elaeagnus conferta | 41.4 | 0.7 | 300.0 | 6 |
| Liana | Elaeagnus conferta | 35.5 | 0.0 | 300.0 | 7 |
| Liana | Elaeagnus conferta | 30.6 | 0.0 | 300.0 | 8 |
| Liana | Elaeagnus conferta | 25.9 | 0.0 | 300.0 | 9 |
| Liana | Elaeagnus conferta | 22.3 | 0.0 | 300.0 | 10 |
| Liana | Maclura cochinchinese | 76.9 | 47.3 | 66.7 | 1 |
| Liana | Maclura cochinchinese | 62.8 | 21.0 | 185.0 | 2 |
| Liana | Maclura cochinchinese | 49.5 | 3.3 | 185.0 | 3 |
| Liana | Maclura cochinchinese | 36.6 | 0.0 | 185.0 | 4 |
| Liana | Maclura cochinchinese | 26.4 | 0.0 | 185.0 | 5 |
| Liana | Maclura cochinchinese | 18.8 | 0.0 | 185.0 | 6 |
| Liana | Maclura cochinchinese | 12.4 | 0.0 | 185.0 | 7 |
| Liana | Maclura cochinchinese | 8.0 | 0.0 | 185.0 | 8 |
| Liana | Maclura cochinchinese | 5.1 | 0.0 | 185.0 | 9 |
| Liana | Maclura cochinchinese | 2.5 | 0.0 | 185.0 | 10 |
| Liana | Olax scandens | 93.6 | 84.0 | 0.0 | 1 |
| Liana | Olax scandens | 89.6 | 74.0 | 46.7 | 2 |
| Liana | Olax scandens | 85.6 | 64.0 | 46.7 | 3 |
| Liana | Olax scandens | 81.6 | 54.0 | 46.7 | 4 |
| Liana | Olax scandens | 77.6 | 44.0 | 46.7 | 5 |
| Liana | Olax scandens | 73.6 | 34.0 | 46.7 | 6 |
| Liana | Olax scandens | 69.6 | 24.0 | 46.7 | 7 |
| Liana | Olax scandens | 65.6 | 14.0 | 46.7 | 8 |
| Liana | Olax scandens | 61.9 | 9.3 | 46.7 | 9 |
| Liana | Olax scandens | 58.2 | 6.0 | 46.7 | 10 |
| Liana | Quisqualis caudata | 76.5 | 68.0 | 41.1 | 1 |
| Liana | Quisqualis caudata | 61.9 | 48.0 | 333.3 | 2 |
| Liana | Quisqualis caudata | 49.3 | 31.3 | 333.3 | 3 |
| Liana | Quisqualis caudata | 39.4 | 19.7 | 333.3 | 4 |
| Liana | Quisqualis caudata | 32.0 | 13.0 | 333.3 | 5 |
| Liana | Quisqualis caudata | 24.8 | 6.3 | 333.3 | 6 |
| Liana | Quisqualis caudata | 18.3 | 0.3 | 333.3 | 7 |
| Liana | Quisqualis caudata | 14.8 | 0.0 | 333.3 | 8 |
| Liana | Quisqualis caudata | 11.4 | 0.0 | 333.3 | 9 |
| Liana | Quisqualis caudata | 8.0 | 0.0 | 333.3 | 10 |
| Prickly | Bombax ceiba | 57.0 | 10.3 | 80.0 | 1 |
| Prickly | Bombax ceiba | 19.9 | 3.3 | 604.4 | 2 |
| Prickly | Bombax ceiba | 2.5 | 0.0 | 1500.0 | 3 |
| Prickly | Bombax ceiba | 0.0 | 0.0 | 2772.7 | 4 |
| Prickly | Bombax ceiba | 0.0 | 0.0 | 4000.0 | 5 |
| Prickly | Bombax ceiba | 0.0 | 0.0 | 6600.0 | 6 |
| Prickly | Bombax ceiba | 0.0 | 0.0 | 8069.2 | 7 |
| Prickly | Bombax ceiba | 0.0 | 0.0 | 11363.6 | 8 |
| Prickly | Bombax ceiba | 0.0 | 0.0 | 14400.0 | 9 |
| Prickly | Bombax ceiba | 0.0 | 0.0 | 18000.0 | 10 |
| Prickly | Ceiba pentandra | 61.6 | 11.3 | 0.0 | 1 |
| Prickly | Ceiba pentandra | 26.9 | 3.0 | 224.4 | 2 |
| Prickly | Ceiba pentandra | 6.1 | 0.0 | 1300.0 | 3 |
| Prickly | Ceiba pentandra | 0.6 | 0.0 | 2231.8 | 4 |
| Prickly | Ceiba pentandra | 0.0 | 0.0 | 3666.7 | 5 |
| Prickly | Ceiba pentandra | 0.0 | 0.0 | 6200.0 | 6 |
| Prickly | Ceiba pentandra | 0.0 | 0.0 | 7684.6 | 7 |
| Prickly | Ceiba pentandra | 0.0 | 0.0 | 9454.5 | 8 |
| Prickly | Ceiba pentandra | 0.0 | 0.0 | 13200.0 | 9 |
| Prickly | Ceiba pentandra | 0.0 | 0.0 | 16000.0 | 10 |
| Prickly | Ceiba speciosa | 59.4 | 0.3 | 33.3 | 1 |
| Prickly | Ceiba speciosa | 23.6 | 0.0 | 491.1 | 2 |
| Prickly | Ceiba speciosa | 4.8 | 0.0 | 900.0 | 3 |
| Prickly | Ceiba speciosa | 0.3 | 0.0 | 2545.5 | 4 |
| Prickly | Ceiba speciosa | 0.0 | 0.0 | 3666.7 | 5 |
| Prickly | Ceiba speciosa | 0.0 | 0.0 | 5800.0 | 6 |
| Prickly | Ceiba speciosa | 0.0 | 0.0 | 7069.2 | 7 |
| Prickly | Ceiba speciosa | 0.0 | 0.0 | 10909.1 | 8 |
| Prickly | Ceiba speciosa | 0.0 | 0.0 | 13200.0 | 9 |
| Prickly | Ceiba speciosa | 0.0 | 0.0 | 18000.0 | 10 |
| Prickly | Hura crepitans | 25.2 | 0.0 | 234.4 | 1 |
| Prickly | Hura crepitans | 1.6 | 0.0 | 1246.7 | 2 |
| Prickly | Hura crepitans | 0.0 | 0.0 | 3100.0 | 3 |
| Prickly | Hura crepitans | 0.0 | 0.0 | 5590.9 | 4 |
| Prickly | Hura crepitans | 0.0 | 0.0 | 9166.7 | 5 |
| Prickly | Hura crepitans | 0.0 | 0.0 | 15000.0 | 6 |
| Prickly | Hura crepitans | 0.0 | 0.0 | 17684.6 | 7 |
| Prickly | Hura crepitans | 0.0 | 0.0 | 25090.9 | 8 |
| Prickly | Hura crepitans | 0.0 | 0.0 | 31800.0 | 9 |
| Prickly | Hura crepitans | 0.0 | 0.0 | 39000.0 | 10 |
| Prickly | Pachira quinata | 27.9 | 0.0 | 170.0 | 1 |
| Prickly | Pachira quinata | 1.5 | 0.0 | 1380.0 | 2 |
| Prickly | Pachira quinata | 0.0 | 0.0 | 2900.0 | 3 |
| Prickly | Pachira quinata | 0.0 | 0.0 | 5863.6 | 4 |
| Prickly | Pachira quinata | 0.0 | 0.0 | 8666.7 | 5 |
| Prickly | Pachira quinata | 0.0 | 0.0 | 15400.0 | 6 |
| Prickly | Pachira quinata | 0.0 | 0.0 | 15400.0 | 7 |
| Prickly | Pachira quinata | 0.0 | 0.0 | 15400.0 | 8 |
| Prickly | Pachira quinata | 0.0 | 0.0 | 15400.0 | 9 |
| Prickly | Pachira quinata | 0.0 | 0.0 | 15400.0 | 10 |
| Prickly | Zanthoxylum armatum | 50.5 | 30.3 | 156.7 | 1 |
| Prickly | Zanthoxylum armatum | 17.8 | 13.3 | 780.0 | 2 |
| Prickly | Zanthoxylum armatum | 5.0 | 5.0 | 2300.0 | 3 |
| Prickly | Zanthoxylum armatum | 1.7 | 1.7 | 3818.2 | 4 |
| Prickly | Zanthoxylum armatum | 0.0 | 0.0 | 5833.3 | 5 |
| Prickly | Zanthoxylum armatum | 0.0 | 0.0 | 9200.0 | 6 |
| Prickly | Zanthoxylum armatum | 0.0 | 0.0 | 11453.8 | 7 |
| Prickly | Zanthoxylum armatum | 0.0 | 0.0 | 13500.0 | 8 |
| Prickly | Zanthoxylum armatum | 0.0 | 0.0 | 13500.0 | 9 |
| Prickly | Zanthoxylum armatum | 0.0 | 0.0 | 13500.0 | 10 |
| Prickly | Zanthoxylum myriacanthum | 70.9 | 0.0 | 24.4 | 1 |
| Prickly | Zanthoxylum myriacanthum | 38.3 | 0.0 | 248.9 | 2 |
| Prickly | Zanthoxylum myriacanthum | 14.0 | 0.0 | 710.0 | 3 |
| Prickly | Zanthoxylum myriacanthum | 3.2 | 0.0 | 1277.3 | 4 |
| Prickly | Zanthoxylum myriacanthum | 0.3 | 0.0 | 2166.7 | 5 |
| Prickly | Zanthoxylum myriacanthum | 0.0 | 0.0 | 3400.0 | 6 |
| Prickly | Zanthoxylum myriacanthum | 0.0 | 0.0 | 3915.4 | 7 |
| Prickly | Zanthoxylum myriacanthum | 0.0 | 0.0 | 5727.3 | 8 |
| Prickly | Zanthoxylum myriacanthum | 0.0 | 0.0 | 8400.0 | 9 |
| Prickly | Zanthoxylum myriacanthum | 0.0 | 0.0 | 11000.0 | 10 |
| Thorny | Alangium salviifolium | 92.5 | 72.3 | 0.0 | 1 |
| Thorny | Alangium salviifolium | 81.0 | 56.7 | 46.7 | 2 |
| Thorny | Alangium salviifolium | 65.6 | 43.3 | 270.0 | 3 |
| Thorny | Alangium salviifolium | 57.6 | 30.3 | 322.7 | 4 |
| Thorny | Alangium salviifolium | 50.7 | 22.0 | 666.7 | 5 |
| Thorny | Alangium salviifolium | 44.2 | 15.3 | 1200.0 | 6 |
| Thorny | Alangium salviifolium | 38.4 | 11.7 | 1200.0 | 7 |
| Thorny | Alangium salviifolium | 32.6 | 8.3 | 1200.0 | 8 |
| Thorny | Alangium salviifolium | 26.9 | 5.0 | 1200.0 | 9 |
| Thorny | Alangium salviifolium | 22.0 | 1.7 | 1200.0 | 10 |
| Thorny | Cassia javanica | 86.1 | 32.3 | 0.0 | 1 |
| Thorny | Cassia javanica | 68.8 | 16.0 | 0.0 | 2 |
| Thorny | Cassia javanica | 50.1 | 5.7 | 270.0 | 3 |
| Thorny | Cassia javanica | 32.8 | 0.3 | 272.7 | 4 |
| Thorny | Cassia javanica | 23.0 | 0.0 | 1000.0 | 5 |
| Thorny | Cassia javanica | 15.4 | 0.0 | 1800.0 | 6 |
| Thorny | Cassia javanica | 10.4 | 0.0 | 2376.9 | 7 |
| Thorny | Cassia javanica | 7.0 | 0.0 | 3000.0 | 8 |
| Thorny | Cassia javanica | 5.2 | 0.0 | 3000.0 | 9 |
| Thorny | Cassia javanica | 4.3 | 0.0 | 3000.0 | 10 |
| Thorny | Citrus lucida | 76.3 | 40.0 | 33.3 | 1 |
| Thorny | Citrus lucida | 54.2 | 26.7 | 284.4 | 2 |
| Thorny | Citrus lucida | 29.8 | 15.7 | 1070.0 | 3 |
| Thorny | Citrus lucida | 11.2 | 6.3 | 1754.5 | 4 |
| Thorny | Citrus lucida | 1.2 | 0.7 | 2833.3 | 5 |
| Thorny | Citrus lucida | 0.0 | 0.0 | 4600.0 | 6 |
| Thorny | Citrus lucida | 0.0 | 0.0 | 5684.6 | 7 |
| Thorny | Citrus lucida | 0.0 | 0.0 | 8454.5 | 8 |
| Thorny | Citrus lucida | 0.0 | 0.0 | 10800.0 | 9 |
| Thorny | Citrus lucida | 0.0 | 0.0 | 13333.3 | 10 |
| Thorny | Cratoxylum cochinchinesis | 80.3 | 25.3 | 0.0 | 1 |
| Thorny | Cratoxylum cochinchinesis | 55.6 | 10.0 | 0.0 | 2 |
| Thorny | Cratoxylum cochinchinesis | 40.6 | 2.3 | 570.0 | 3 |
| Thorny | Cratoxylum cochinchinesis | 29.9 | 0.0 | 1045.5 | 4 |
| Thorny | Cratoxylum cochinchinesis | 21.9 | 0.0 | 1800.0 | 5 |
| Thorny | Cratoxylum cochinchinesis | 15.5 | 0.0 | 1800.0 | 6 |
| Thorny | Cratoxylum cochinchinesis | 10.9 | 0.0 | 1800.0 | 7 |
| Thorny | Cratoxylum cochinchinesis | 6.8 | 0.0 | 1800.0 | 8 |
| Thorny | Cratoxylum cochinchinesis | 4.0 | 0.0 | 1800.0 | 9 |
| Thorny | Cratoxylum cochinchinesis | 2.6 | 0.0 | 1800.0 | 10 |
| Thorny | Flacourtia indica | 71.9 | 27.3 | 0.0 | 1 |
| Thorny | Flacourtia indica | 51.5 | 17.3 | 180.0 | 2 |
| Thorny | Flacourtia indica | 30.7 | 7.3 | 803.3 | 3 |
| Thorny | Flacourtia indica | 11.5 | 2.3 | 1913.6 | 4 |
| Thorny | Flacourtia indica | 5.5 | 0.0 | 3283.3 | 5 |
| Thorny | Flacourtia indica | 2.1 | 0.0 | 6600.0 | 6 |
| Thorny | Flacourtia indica | 1.1 | 0.0 | 7684.6 | 7 |
| Thorny | Flacourtia indica | 0.6 | 0.0 | 12454.5 | 8 |
| Thorny | Flacourtia indica | 0.3 | 0.0 | 12454.5 | 9 |
| Thorny | Flacourtia indica | 0.0 | 0.0 | 12454.5 | 10 |
| Thorny | Flacourtia rukam | 79.7 | 24.0 | 15.6 | 1 |
| Thorny | Flacourtia rukam | 58.0 | 9.7 | 133.3 | 2 |
| Thorny | Flacourtia rukam | 36.6 | 5.0 | 300.0 | 3 |
| Thorny | Flacourtia rukam | 25.8 | 1.7 | 818.2 | 4 |
| Thorny | Flacourtia rukam | 18.6 | 0.0 | 1500.0 | 5 |
| Thorny | Flacourtia rukam | 14.0 | 0.0 | 3000.0 | 6 |
| Thorny | Flacourtia rukam | 10.7 | 0.0 | 3000.0 | 7 |
| Thorny | Flacourtia rukam | 8.1 | 0.0 | 3000.0 | 8 |
| Thorny | Flacourtia rukam | 6.4 | 0.0 | 3000.0 | 9 |
| Thorny | Flacourtia rukam | 4.7 | 0.0 | 3000.0 | 10 |
| Thorny | Gleditsia microphylla | 19.2 | 6.0 | 412.2 | 1 |
| Thorny | Gleditsia microphylla | 4.1 | 2.7 | 2646.7 | 2 |
| Thorny | Gleditsia microphylla | 0.1 | 0.0 | 5540.0 | 3 |
| Thorny | Gleditsia microphylla | 0.0 | 0.0 | 11136.4 | 4 |
| Thorny | Gleditsia microphylla | 0.0 | 0.0 | 16500.0 | 5 |
| Thorny | Gleditsia microphylla | 0.0 | 0.0 | 25400.0 | 6 |
| Thorny | Gleditsia microphylla | 0.0 | 0.0 | 25400.0 | 7 |
| Thorny | Gleditsia microphylla | 0.0 | 0.0 | 25400.0 | 8 |
| Thorny | Gleditsia microphylla | 0.0 | 0.0 | 25400.0 | 9 |
| Thorny | Gleditsia microphylla | 0.0 | 0.0 | 25400.0 | 10 |

Table S8: Predictive posterior parameters and Bayesian goodness-of-fit for the Bayesian models of computer simulation analyses (N_Liana_= 8 sp., N_Crown_= 9 sp., N_Thorny_= 7 sp., N_Prickly_= 7 sp.).

|  | **Estimate** | **Est.Error** | **l-95% CI** | **u-95% CI** | **Rhat** |
| --- | --- | --- | --- | --- | --- |
| *Bark proportion ~ Size:Group + (1|Observation)* | | | | |  |
| ***Population-Level Effects*** |  |  |  |  |  |
| Intercept | 1.33 | 0.18 | 0.97 | 1.69 | 1.00 |
| Liana | -0.43 | 0.04 | -0.50 | -0.35 | 1.00 |
| Crown | -0.37 | 0.04 | -0.44 | -0.30 | 1.00 |
| Thorny | -0.60 | 0.04 | -0.68 | -0.52 | 1.00 |
| Prickly | -1.71 | 0.12 | -1.94 | -1.49 | 1.00 |
| ***Group-Level Effects*** |  |  |  |  |  |
| Observation | 1.32 | 0.07 | 1.19 | 1.47 | 1.00 |
| ***Goodness-of-fit*** |  |  |  |  |  |
| R² | 0.984 |  | 0.981 | 0.987 |  |
|  |  |  |  |  |  |
| *Ring probability ~ Size:Group + (1|Observation)* | | |  |  |  |
| ***Population-Level Effects*** |  |  |  |  |  |
| Intercept | 0.44 | 0.33 | -0.21 | 1.11 | 1.00 |
| Liana | -0.89 | 0.09 | -1.07 | -0.74 | 1.00 |
| Crown | -0.94 | 0.09 | -1.13 | -0.78 | 1.00 |
| Thorny | -1.01 | 0.09 | -1.21 | -0.84 | 1.00 |
| Prickly | -2.56 | 0.29 | -3.15 | -2.02 | 1.00 |
| ***Group-Level Effects*** |  |  |  |  |  |
| Observation | 2.18 | 0.17 | 1.88 | 2.55 | 1.00 |
| ***Goodness-of-fit*** |  |  |  |  |  |
| R² | 0.983 |  | 0.978 | 0.988 |  |
|  |  |  |  |  |  |
| *Slowdown ~ Size:Group + (1|Observation)* | | | | |  |
| ***Population-Level Effects*** | |  |  |  |  |
| Intercept | 4.38 | 0.17 | 4.05 | 4.71 | 1.00 |
| Liana | 0.19 | 0.03 | 0.12 | 0.26 | 1.00 |
| Crown | 0.17 | 0.03 | 0.11 | 0.24 | 1.00 |
| Thorny | 0.50 | 0.04 | 0.43 | 0.57 | 1.00 |
| Prickly | 0.65 | 0.04 | 0.58 | 0.72 | 1.00 |
| ***Group-Level Effects*** | |  |  |  |  |
| Observation | 1.36 | 0.06 | 1.24 | 1.49 | 1.00 |
| ***Goodness-of-fit*** |  |  |  |  |  |
| R² | 0.999 | 0.001 | 0.999 | 1.000 |  |

Table S9: General non-linear hypothesis test of the Bayesian models for the computer simulation analyses (N_Liana_= 8 sp., N_Crown_= 9 sp., N_Thorny_= 7 sp., N_Prickly_= 7 sp.).

| **Hypothesis** | **Estimate** | **Est.Error** | **l-95% CI** | **u-95% CI** | **Evid.Ratio** | **Post.Prob** | **Star** |
| --- | --- | --- | --- | --- | --- | --- | --- |
| *Bark proportion ~ size:group + (1|Observation)* | | | | | | | |
| Liana = Crown | 0.05 | 0.03 | -0.02 | 0.12 | 51.95 | 0.98 |  |
| Liana = Thorny | 0.17 | 0.04 | 0.10 | 0.24 | 0.00 | 0.00 | * |
| Liana = Prickly | 1.28 | 0.11 | 1.08 | 1.50 | 0.00 | 0.00 | * |
| Crown = Thorny | 0.22 | 0.04 | 0.15 | 0.29 | 0.00 | 0.00 | * |
| Crown = Prickly | 1.33 | 0.11 | 1.13 | 1.55 | 0.00 | 0.00 | * |
| Prickly = Thorny | -1.11 | 0.11 | -1.33 | -0.90 | 0.00 | 0.00 | * |
|  |  |  |  |  |  |  |  |
| *Ring probability ~ Size:Group + (1|Observation)* | | | | | | | |
| Liana = Crown | -0.05 | 0.08 | -0.21 | 0.11 | 63.79 | 0.98 |  |
| Liana = Thorny | 0.12 | 0.09 | -0.05 | 0.29 | 28.16 | 0.97 |  |
| Liana = Prickly | 1.66 | 0.27 | 1.17 | 2.21 | 0.00 | 0.00 | * |
| Crown = Thorny | 0.07 | 0.09 | -0.11 | 0.25 | 49.87 | 0.98 |  |
| Crown = Prickly | 1.61 | 0.27 | 1.12 | 2.16 | 0.00 | 0.00 | * |
| Prickly = Thorny | -1.55 | 0.27 | -2.09 | -1.04 | 0.00 | 0.00 | * |
|  |  |  |  |  |  |  |  |
| *Slowdown ~ Size:Group + (1|Observation)* | | | | | | | |
| Liana = Crown | -0.02 | 0.03 | -0.09 | 0.05 | 85.26 | 0.99 |  |
| Liana = Thorny | -0.31 | 0.04 | -0.38 | -0.24 | 0.00 | 0.00 | * |
| Liana = Prickly | -0.45 | 0.04 | -0.53 | -0.38 | 0.00 | 0.00 | * |
| Crown = Thorny | -0.33 | 0.04 | -0.40 | -0.26 | 0.00 | 0.00 | * |
| Crown = Prickly | -0.47 | 0.03 | -0.54 | -0.40 | 0.00 | 0.00 | * |
| Prickly = Thorny | 0.15 | 0.04 | 0.08 | 0.22 | 0.04 | 0.00 | * |
| ** indicates a significant difference base on 95%CI* | | | | |  |  |  |

Table S10: Bayesian model selection for the nutritional analyses using “Leave-One-Out cross-validation Information Criterion” method.

| **Response** | **LOOIC (± SE)** | **Right side of the formula** |
| --- | --- | --- |
| *Inner bark* | | |
| Total phenol | 460.5 (18.6) | 0 + Syndrome + (1|Phylogeny) + (1|Species/Month) |
|  | 460.0 (19.4) | 0 + Syndrome + (1|Phylogeny) + (1|Species) |
|  | 460.1 (18.8) | 0 + Syndrome + (1|Species/Month) |
|  | **456.8 (19.1)** | 0 + Syndrome + (1|Species) |
|  | 464.5 (19.2) | 0 + Syndrome + (1|Phylogeny) |
|  | 521.5 (17.6) | 0 + Syndrome |
| Nitrogen | 52.6 (21.7) | 0 + Syndrome + (1|Phylogeny) + (1|Species/Month) |
|  | 54.5 (23.7) | 0 + Syndrome + (1|Phylogeny) + (1|Species) |
|  | **50.3 (21.6)** | 0 + Syndrome + (1|Species/Month) |
|  | 51.5 (23.5) | 0 + Syndrome + (1|Species) |
|  | 66.1 (21.8) | 0 + Syndrome + (1|Phylogeny) |
|  | 105.0 (18.4) | 0 + Syndrome |
| Inner bark thickness | **20.1 (29.3)** | 0 + Syndrome + (1|Phylogeny) + (1|Species) |
|  | 21.5 (29.9) | 0 + Syndrome + (1|Phylogeny) |
|  | 33.6 (29.0) | 0 + Syndrome + (1|Species) |
|  | 100.7 (23.0) | 0 + Syndrome |
|  |  |  |
| *Leaf* | | |
| Total phenol | **316.4 (30.3)** | 0 + Syndrome + (1|Phylogeny) + (1|Species) |
|  | 318.9 (29.6) | 0 + Syndrome + (1|Phylogeny) |
|  | 317.1 (30.4) | 0 + Syndrome + (1|Species) |
|  | 423.8 (21.2) | 0 + Syndrome |
| Nitrogen | 197.0 (32.8) | 0 + Syndrome + (1|Phylogeny) + (1|Species) |
|  | 211.5 (27.8) | 0 + Syndrome + (1|Phylogeny) |
|  | **196.5 (32.8)** | 0 + Syndrome + (1|Species) |
|  | 227.7 (16.3) | 0 + Syndrome |

Table S11: Predictive posterior parameters and Bayesian goodness-of-fit for the Bayesian models of nutritional analyses.

| *Inner bark* |  |  |  |  |  |  | *Leaf* | | | | |  |
| --- | --- | --- | --- | --- | --- | --- | --- | --- | --- | --- | --- | --- |
|  | **Estimate** | **Est.Error** | **l-95% CI** | **u-95% CI** | **Rhat** |  |  | **Estimate** | **Est.Error** | **l-95% CI** | **u-95% CI** | **Rhat** |
| *Total phenol ~ 0 + Syndrome + (1|Species)* | | | | |  |  | *Total phenol ~ 0 + Syndrome + (1|Phylogeny) + (1|Species)* | | | | |  |
| ***Population-Level Effects*** |  |  |  |  |  |  | ***Population-Level Effects*** |  |  |  |  |  |
| Liana | 1.24 | 0.29 | 0.66 | 1.79 | 1.00 |  | Liana | 1.56 | 0.37 | 0.76 | 2.25 | 1.00 |
| Crown | 1.18 | 0.26 | 0.68 | 1.68 | 1.00 |  | Crown | 1.02 | 0.34 | 0.26 | 1.64 | 1.00 |
| Non-spiny | 1.35 | 0.16 | 1.03 | 1.68 | 1.00 |  | Non-spiny | 0.96 | 0.29 | 0.34 | 1.49 | 1.00 |
| Thorny | 1.38 | 0.29 | 0.81 | 1.95 | 1.00 |  | Thorny | 1.42 | 0.37 | 0.64 | 2.10 | 1.00 |
| Prickly | 1.03 | 0.31 | 0.42 | 1.64 | 1.00 |  | Prickly | 0.34 | 0.38 | -0.45 | 1.07 | 1.00 |
| ***Group-Level Effects*** |  |  |  |  |  |  | ***Group-Level Effects*** |  |  |  |  |  |
| Species | 0.68 | 0.09 | 0.52 | 0.88 | 1.00 |  | Species | 0.64 | 0.10 | 0.46 | 0.86 | 1.00 |
| ***Family specific Parameters*** |  |  |  |  |  |  | Phylogeny | 0.34 | 0.28 | 0.01 | 1.07 | 1.00 |
| Sigma | 0.44 | 0.04 | 0.36 | 0.54 | 1.00 |  | ***Family Specific Parameters*** |  |  |  |  |  |
| ***Goodness-of-fit*** |  |  |  |  |  |  | Sigma | 0.32 | 0.03 | 0.26 | 0.38 | 1.00 |
| R² | 0.693 | 0.063 | 0.549 | 0.791 |  |  | ***Goodness-of-fit*** |  |  |  |  |  |
|  |  |  |  |  |  |  | R² | 0.824 | 0.044 | 0.714 | 0.885 |  |
|  |  |  |  |  |  |  |  |  |  |  |  |  |
| *Nitrogen ~ 0 + Syndrome + (1|Species/Month)* | | | | |  |  | *Nitrogen ~ 0 + Syndrome + (1|Species)* | | | | |  |
| ***Population-Level Effects*** |  |  |  |  |  |  | ***Population-Level Effects*** |  |  |  |  |  |
| Liana | 0.24 | 0.12 | 0.00 | 0.48 | 1.00 |  | Liana | 0.96 | 0.12 | 0.72 | 1.20 | 1.00 |
| Crown | 0.12 | 0.11 | -0.09 | 0.34 | 1.00 |  | Crown | 0.66 | 0.11 | 0.44 | 0.87 | 1.00 |
| Non-spiny | 0.15 | 0.07 | 0.01 | 0.28 | 1.00 |  | Non-spiny | 0.69 | 0.08 | 0.53 | 0.86 | 1.00 |
| Thorny | 0.10 | 0.12 | -0.14 | 0.33 | 1.00 |  | Thorny | 0.89 | 0.13 | 0.64 | 1.14 | 1.00 |
| Prickly | -0.10 | 0.13 | -0.36 | 0.16 | 1.00 |  | Prickly | 0.76 | 0.12 | 0.51 | 1.00 | 1.00 |
| ***Group-Level Effects*** |  |  |  |  |  |  | ***Group-Level Effects*** |  |  |  |  |  |
| Species | 0.28 | 0.04 | 0.20 | 0.37 | 1.00 |  | Species | 0.26 | 0.05 | 0.18 | 0.36 | 1.00 |
| Species:Month | 0.07 | 0.05 | 0.00 | 0.18 | 1.00 |  | ***Family Specific Parameters*** |  |  |  |  |  |
| ***Family specific Parameters*** |  |  |  |  |  |  | Sigma | 0.24 | 0.02 | 0.20 | 0.29 | 1.00 |
| Sigma | 0.20 | 0.02 | 0.16 | 0.24 | 1.00 |  | ***Goodness-of-fit*** |  |  |  |  |  |
| ***Goodness-of-fit*** |  |  |  |  |  |  | R² | 0.622 | 0.077 | 0.442 | 0.739 |  |
| R² | 0.688 | 0.067 | 0.538 | 0.802 |  |  |  |  |  |  |  |  |
|  |  |  |  |  |  |  |  |  |  |  |  |  |
| *Inner bark thickness ~ 0 + Syndrome + (1|Phylogeny) + (1|Species)* | | | |  |  |  |  |  |  |  |  |  |
| ***Population-Level Effects*** |  |  |  |  |  |  |  |  |  |  |  |  |
| Liana | -0.01 | 0.12 | -0.26 | 0.23 | 1.00 |  |  |  |  |  |  |  |
| Crown | 0.08 | 0.11 | -0.13 | 0.30 | 1.00 |  |  |  |  |  |  |  |
| Non-spiny | 0.24 | 0.08 | 0.08 | 0.39 | 1.00 |  |  |  |  |  |  |  |
| Thorny | 0.03 | 0.12 | -0.22 | 0.25 | 1.00 |  |  |  |  |  |  |  |
| Prickly | 0.88 | 0.14 | 0.58 | 1.14 | 1.00 |  |  |  |  |  |  |  |
| ***Group-Level Effects*** |  |  |  |  |  |  |  |  |  |  |  |  |
| Species | 0.24 | 0.04 | 0.16 | 0.31 | 1.00 |  |  |  |  |  |  |  |
| Phylogeny | 0.11 | 0.07 | 0.01 | 0.28 | 1.00 |  |  |  |  |  |  |  |
| ***Family specific Parameters*** |  |  |  |  |  |  |  |  |  |  |  |  |
| Sigma | 0.15 | 0.02 | 0.12 | 0.18 | 1.00 |  |  |  |  |  |  |  |
| ***Goodness-of-fit*** |  |  |  |  |  |  |  |  |  |  |  |  |
| R² | 0.898 | 0.023 | 0.840 | 0.927 |  |  |  |  |  |  |  |  |

Table S12: General non-linear hypothesis test of the Bayesian models for the nutritional analyses.

| *Inner bark* |  |  |  |  |  |  |  |  | *Leaf* |  |  |  |  |  |  |  |
| --- | --- | --- | --- | --- | --- | --- | --- | --- | --- | --- | --- | --- | --- | --- | --- | --- |
| **Hypothesis** | **Estimate** | **Est.Error** | **l-95% CI** | **u-95% CI** | **Evid.Ratio** | **Post.Prob** | **Star** |  | **Hypothesis** | **Estimate** | **Est.Error** | **l-95% CI** | **u-95% CI** | **Evid.Ratio** | **Post.Prob** | **Star** |
| *Total phenol ~ 0 + Syndrome + (1|Species)* | | | | |  |  |  |  | *Total phenol ~ 0 + Syndrome + (1|Phylogeny) + (1|Species)* | | | | | |  |  |
| Liana = Crown | 0.06 | 0.38 | -0.70 | 0.80 | 8.21 | 0.89 |  |  | Liana = Crown | 0.54 | 0.40 | -0.24 | 1.33 | 2.96 | 0.75 |  |
| Liana = Thorny | -0.14 | 0.40 | -0.95 | 0.64 | 7.12 | 0.88 |  |  | Liana = Thorny | 0.14 | 0.41 | -0.67 | 0.93 | 6.49 | 0.87 |  |
| Liana = Prickly | 0.20 | 0.42 | -0.64 | 1.03 | 6.60 | 0.87 |  |  | Liana = Prickly | 1.22 | 0.43 | 0.35 | 2.04 | 0.16 | 0.14 | * |
| Liana = Non-spiny | -0.12 | 0.33 | -0.76 | 0.52 | 8.76 | 0.90 |  |  | Liana = Non-spiny | 0.60 | 0.34 | -0.10 | 1.27 | 1.72 | 0.63 |  |
| Crown = Thorny | -0.20 | 0.38 | -0.94 | 0.57 | 7.25 | 0.88 |  |  | Crown = Thorny | -0.40 | 0.39 | -1.18 | 0.37 | 4.39 | 0.81 |  |
| Crown = Prickly | 0.15 | 0.40 | -0.64 | 0.93 | 6.93 | 0.87 |  |  | Crown = Prickly | 0.68 | 0.42 | -0.15 | 1.50 | 1.92 | 0.66 |  |
| Crown = Non-spiny | -0.17 | 0.30 | -0.78 | 0.42 | 8.55 | 0.90 |  |  | Crown = Non-spiny | 0.06 | 0.32 | -0.57 | 0.69 | 8.59 | 0.90 |  |
| Thorny = Prickly | 0.35 | 0.42 | -0.49 | 1.17 | 5.57 | 0.85 |  |  | Thorny = Prickly | 1.08 | 0.42 | 0.23 | 1.90 | 0.30 | 0.23 | * |
| Thorny = Non-spiny | 0.03 | 0.33 | -0.62 | 0.67 | 9.65 | 0.91 |  |  | Thorny = Non-spiny | 0.46 | 0.34 | -0.23 | 1.12 | 3.39 | 0.77 |  |
| Non-spiny = Prickly | -0.32 | 0.35 | -1.01 | 0.37 | 6.02 | 0.86 |  |  | Prickly = Non-spiny | -0.62 | 0.36 | -1.34 | 0.09 | 1.75 | 0.64 |  |
|  |  |  |  |  |  |  |  |  |  |  |  |  |  |  |  |  |
| *Nitrogen ~ 0 + Syndrome + (1|Species/Month)* | | | | |  |  |  |  | *Nitrogen ~ 0 + Syndrome + (1|Species)* | | | |  |  |  |  |
| Liana = Crown | 0.12 | 0.16 | -0.20 | 0.44 | 11.16 | 0.92 |  |  | Liana = Crown | 0.30 | 0.16 | -0.02 | 0.63 | 2.07 | 0.67 |  |
| Liana = Thorny | 0.14 | 0.17 | -0.20 | 0.48 | 9.92 | 0.91 |  |  | Liana = Thorny | 0.07 | 0.17 | -0.27 | 0.42 | 9.51 | 0.90 |  |
| Liana = Prickly | 0.34 | 0.18 | -0.01 | 0.69 | 2.19 | 0.69 |  |  | Liana = Prickly | 0.21 | 0.17 | -0.14 | 0.55 | 5.90 | 0.86 |  |
| Liana = Non-spiny | 0.10 | 0.14 | -0.18 | 0.37 | 12.73 | 0.93 |  |  | Liana = Non-spiny | 0.27 | 0.15 | -0.02 | 0.55 | 2.53 | 0.72 |  |
| Crown = Thorny | 0.02 | 0.16 | -0.30 | 0.34 | 13.79 | 0.93 |  |  | Crown = Thorny | -0.23 | 0.16 | -0.56 | 0.09 | 4.28 | 0.81 |  |
| Crown = Prickly | 0.22 | 0.17 | -0.12 | 0.56 | 6.41 | 0.87 |  |  | Crown = Prickly | -0.10 | 0.16 | -0.42 | 0.23 | 11.00 | 0.92 |  |
| Crown = Non-spiny | -0.03 | 0.13 | -0.28 | 0.23 | 16.24 | 0.94 |  |  | Crown = Non-spiny | -0.04 | 0.14 | -0.31 | 0.23 | 14.89 | 0.94 |  |
| Thorny = Prickly | 0.20 | 0.18 | -0.15 | 0.54 | 6.40 | 0.86 |  |  | Thorny = Prickly | 0.13 | 0.18 | -0.22 | 0.48 | 8.39 | 0.89 |  |
| Thorny = Non-spiny | -0.05 | 0.14 | -0.33 | 0.22 | 14.91 | 0.94 |  |  | Thorny = Non-spiny | 0.19 | 0.15 | -0.10 | 0.49 | 5.26 | 0.84 |  |
| Non-spiny = Prickly | -0.25 | 0.15 | -0.54 | 0.04 | 4.00 | 0.80 |  |  | Prickly = Non-spiny | 0.06 | 0.15 | -0.24 | 0.35 | 13.23 | 0.93 |  |
|  |  |  |  |  |  |  |  |  |  | | | |  |  |  |  |
| *Inner bark thickness ~ 0 + Syndrome + (1|Phylogeny) + (1|Species)* | | | | |  |  |  |  |  |  |  |  |  |  |  |  |
| Liana = Crown | -0.09 | 0.15 | -0.39 | 0.20 | 9.67 | 0.91 |  |  |  |  |  |  |  |  |  |  |
| Liana = Thorny | -0.04 | 0.16 | -0.34 | 0.27 | 11.21 | 0.92 |  |  |  |  |  |  |  |  |  |  |
| Liana = Prickly | -0.89 | 0.18 | -1.23 | -0.54 | 0.00 | 0.00 | * |  |  |  |  |  |  |  |  |  |
| Liana = Non-spiny | -0.25 | 0.13 | -0.50 | -0.01 | 1.90 | 0.66 | * |  |  |  |  |  |  |  |  |  |
| Crown = Thorny | 0.05 | 0.15 | -0.24 | 0.35 | 9.53 | 0.91 |  |  |  |  |  |  |  |  |  |  |
| Crown = Prickly | -0.80 | 0.17 | -1.12 | -0.46 | 0.00 | 0.00 | * |  |  |  |  |  |  |  |  |  |
| Crown = Non-spiny | -0.16 | 0.11 | -0.38 | 0.07 | 5.91 | 0.86 |  |  |  |  |  |  |  |  |  |  |
| Thorny = Prickly | -0.85 | 0.17 | -1.17 | -0.52 | 0.00 | 0.00 | * |  |  |  |  |  |  |  |  |  |
| Thorny = Non-spiny | -0.21 | 0.13 | -0.47 | 0.04 | 2.39 | 0.70 |  |  |  |  |  |  |  |  |  |  |
| Non-spiny = Prickly | 0.64 | 0.14 | 0.35 | 0.92 | 0.00 | 0.00 | * |  |  |  |  |  |  |  |  |  |
| ** Indicates a significant difference based on 95%CI* | | | |  |  |  |  |  |  |  |  |  |  |  |  |  |

Table S13: Predicted attractiveness of fruit and flower for the 31 spiny species.

| **Species** | **Syndrome** | **Fruit** | **Fleur** |
| --- | --- | --- | --- |
| Ceiba pentandra | Prickly | Low | Large |
| Ceiba speciosa | Prickly | Low | Large |
| Bombax ceiba | Prickly | Low | Large |
| Pachira quinata | Prickly | Low | Large |
| Hura crepitans | Prickly | Low | Large |
| Zanthoxylum myriacanthum | Prickly | Low | Small |
| Zanthoxylum armatum | Prickly | Low | Small |
| Gleditsia microphylla | Thorny | Medium | Small |
| Flacourtia indica | Thorny | High | Small |
| Flacourtia rukam | Thorny | High | Small |
| Cratoxylum cochinchinense | Thorny | Low | Small |
| Citrus lucida | Thorny | High | Small |
| Cassia javanica | Thorny | Medium | Large |
| Alangium salviifolium | Thorny | High | Small |
| Artabotrys hexapetalus | Crown | High | Large |
| Sapium glandulosum | Crown | Low | Small |
| Maytenus austroyunnanensis | Crown | Low | Small |
| Gmelina philippensis | Crown | High | Large |
| Maytenus hookeri | Crown | Low | Small |
| Paliurus ramosissimus | Crown | Low | Small |
| Rhamnella wilsonii | Crown | Low | Small |
| Gmelina asiatica | Crown | High | Large |
| Catunaregam spinosa | Crown | High | Small |
| Olax scandens | Liana | High | Small |
| Combretum indicum | Liana | Low | Large |
| Quisqualis caudata | Liana | Low | Large |
| Maclura cochinchinensis | Liana | High | Small |
| Acacia pennata | Liana | Medium | Small |
| Caesalpinia cucullata | Liana | Medium | Small |
| Caesalpinia minax | Liana | Medium | Small |
| Elaeagnus conferta | Liana | High | Small |


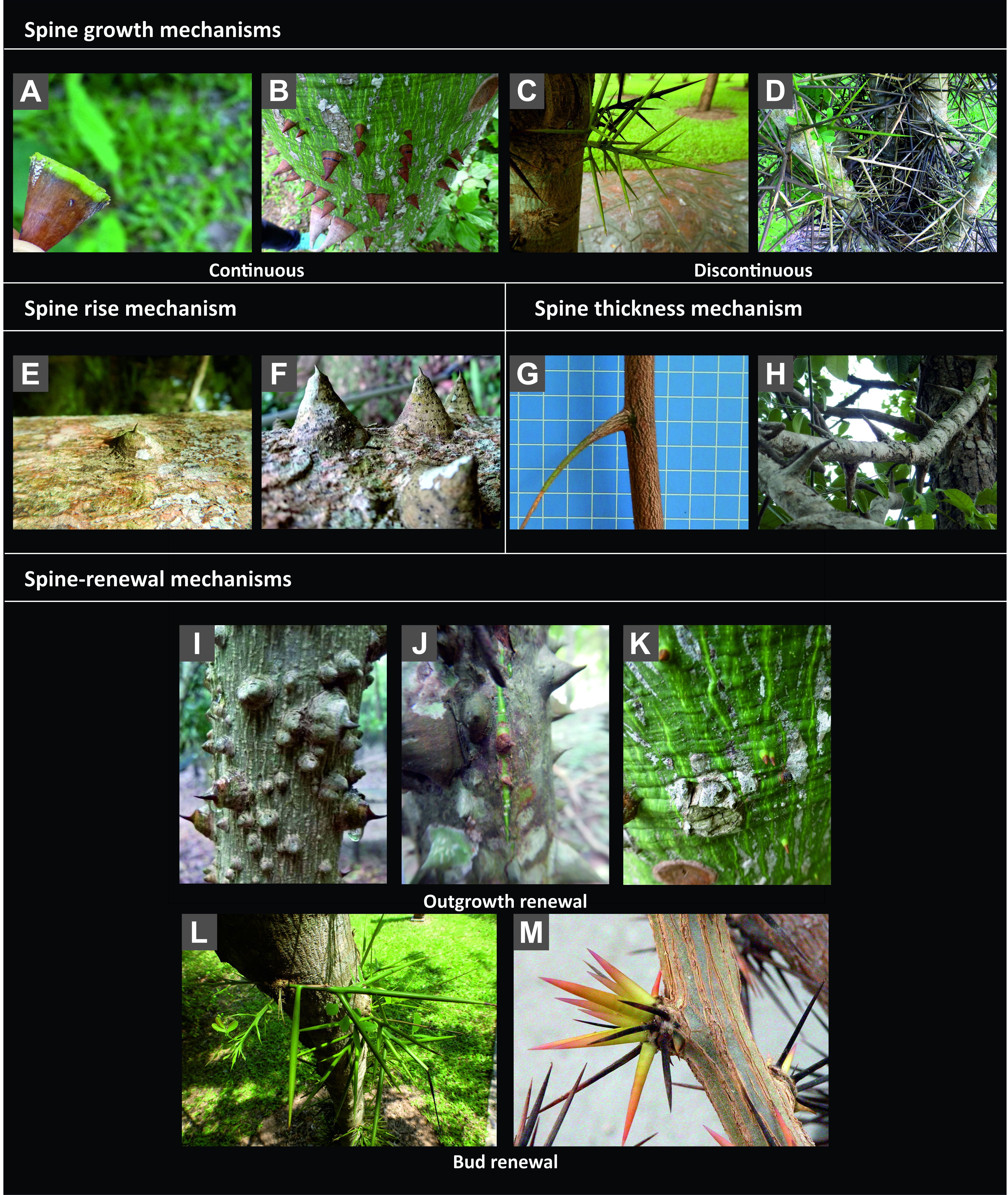


Figure S1: Photographs of spine variables. (A) and (B) basal continuous growth of cork spines from *Ceiba speciosa*, with spines that growth and hardened at the same time. (C) and (D) discontinuous establishment of thorns from *Gleditsia microphylla*, with a first step where the spine is fully established (in green), and a second step during which the spine is hardened. (E) and (F) basal rising of prickles from *Zanthoxylum myriacanthum*. (G) and (H) lateral thickening of thorns from *Olax scandens*. (I), (J) and (K) late recruitment of cork spines on old trunk from *Zanthoxylum* sp., *Pachira quinata* and *Ceiba speciosa*, respectively. (I) both prickles and cork spines are observable on the trunk, with the first one being set with a sharp spine tip. (L) and (M) late recruitment of thorns and leaf spines from *Gleditsia microphylla* and *Pereskia aculeata* (picture from Dieter Helm), respectively.


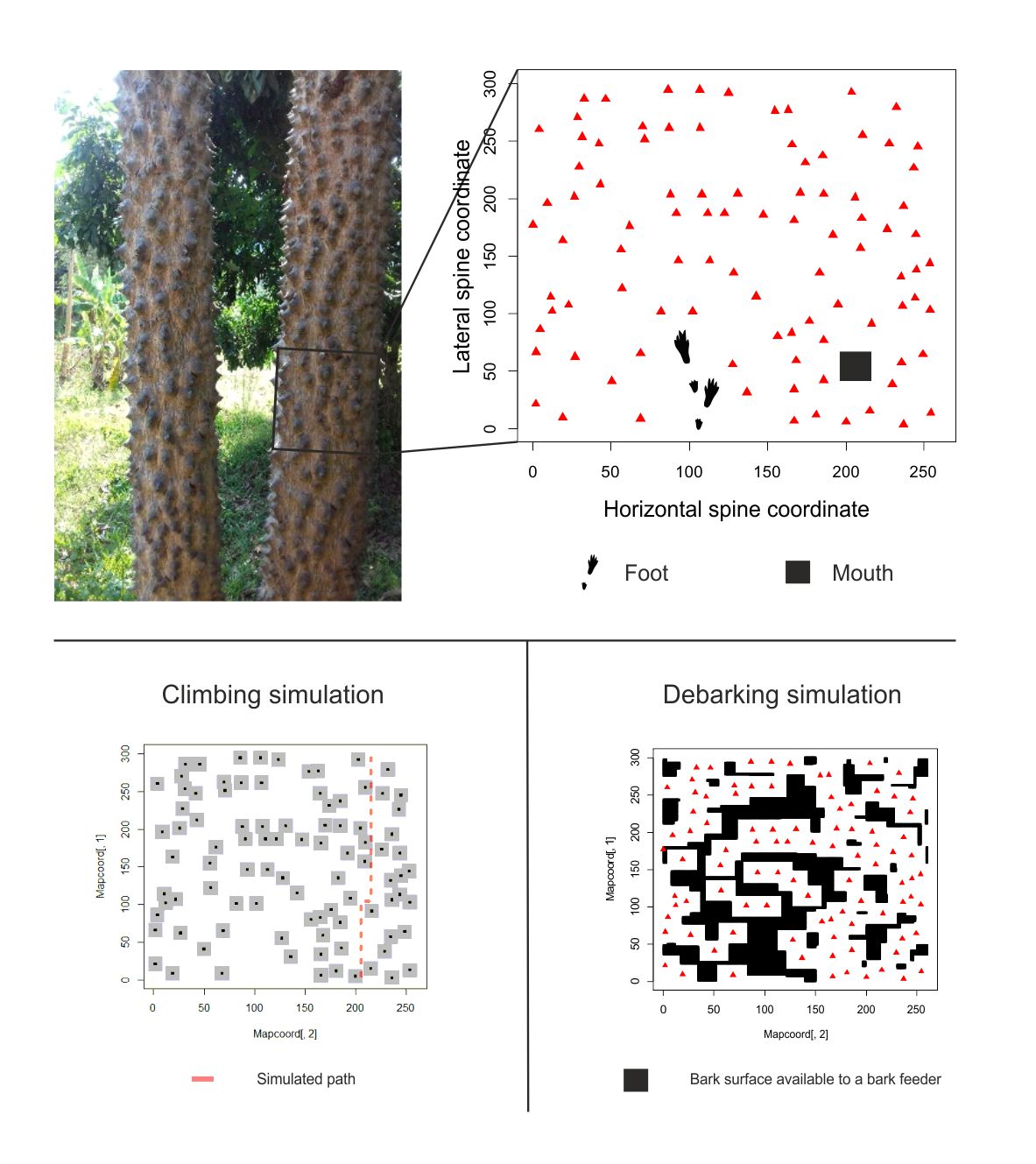


Figure S2: Simplified graphical representation of the computer simulation methodology about mammal debarking and climbing on spiny trunk species.


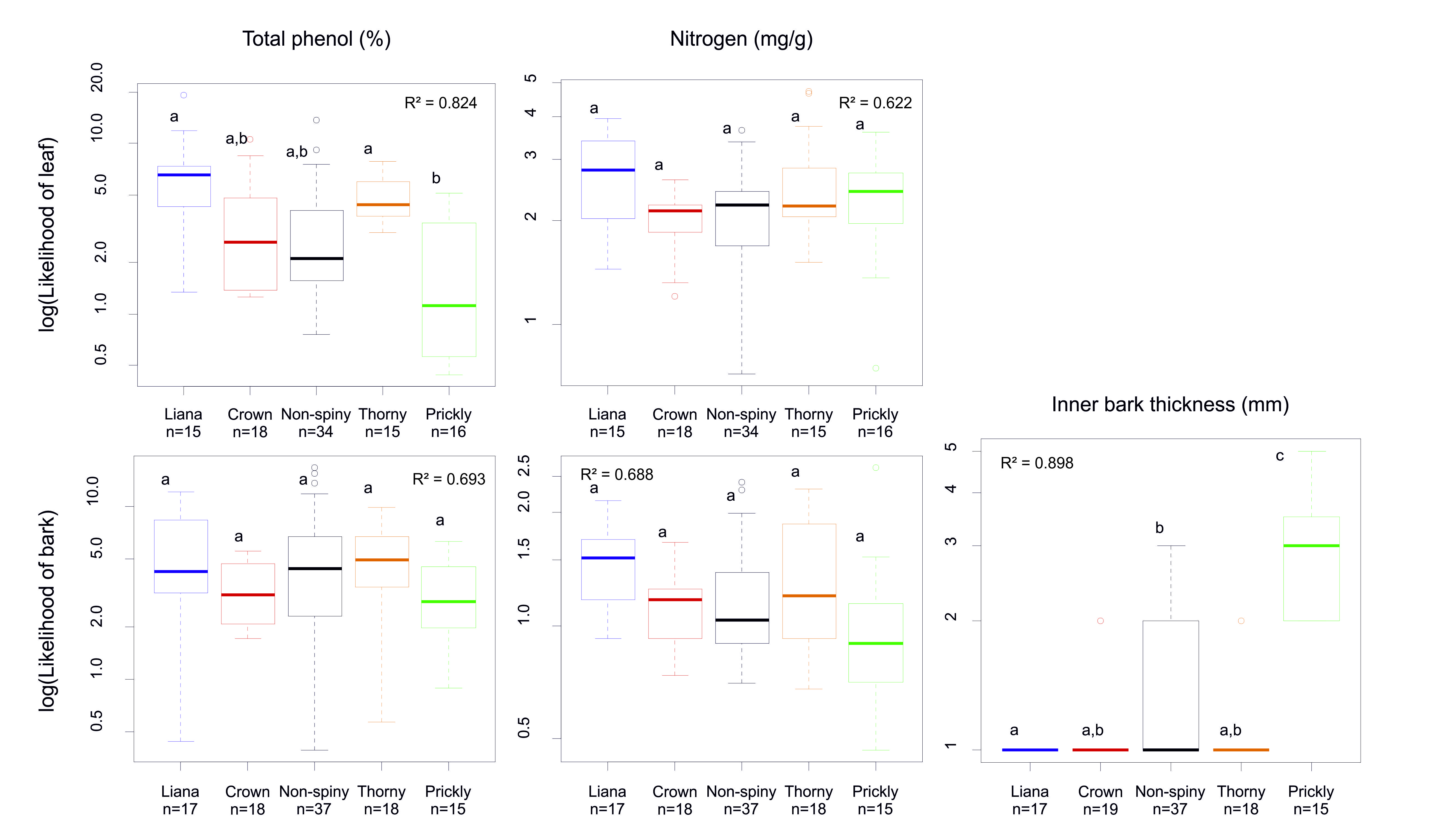


Figure S3: Median estimates of total phenol and nitrogen in leaves and inner bark, as well as inner bark thickness, for each spiny syndrome and for a non-spiny group with confamilial species (N_Liana_= 7 sp., N_Crown_= 9 sp., N_Thorny_= 7 sp., N_Prickly_= 6 sp., N_Non-spiny_= 27 sp.). Values were log transformed. Posterior predictive parameters estimated from the Bayesian model parameters and multiple comparison parameters estimated are referenced in the table S9 and S10. Significant letters are based on 95%CI.
